# Drivers of population differentiation in phenotypic plasticity in a temperate conifer: A 27‐year study

**DOI:** 10.1111/eva.13492

**Published:** 2022-10-31

**Authors:** Raul de la Mata, Rafael Zas, Gloria Bustingorri, Luis Sampedro, Marc Rust, Ana Hernandez‐Serrano, Anna Sala

**Affiliations:** ^1^ Division of Biological Sciences University of Montana Missoula Montana USA; ^2^ Estación Biológica de Doñana Consejo Superior de Investigaciones Científicas (EBD‐CSIC) Sevilla Spain; ^3^ Misión Biológica de Galicia Consejo Superior de Investigaciones Científicas (MBG‐CSIC) Pontevedra Spain; ^4^ Inland Empire Tree Improvement Cooperative University of Idaho Moscow Idaho USA; ^5^ Centre de Recerca Ecològica i Aplicacions Forestals (CREAF) Cerdanyola del Vallès Spain

**Keywords:** environmental heterogeneity, genotype by environment interaction, genotypic stability, patch size, phenotypic plasticity, *Pinus ponderosa*

## Abstract

Phenotypic plasticity is a main mechanism for organisms to cope with changing environments and broaden their ecological range. Plasticity is genetically based and can evolve under natural selection, such that populations within a species show distinct phenotypic responses to the environment if evolved under different conditions. Understanding how intraspecific variation in phenotypic plasticity arises is critical to assess potential adaptation to ongoing climate change. Theory predicts that plasticity is favored in more favorable but variable environments. Yet, many theoretical predictions about benefits, costs, and selection on plasticity remain untested. To test these predictions, we took advantage of three genetic trials in the northern Rocky Mountains, USA, which assessed 23 closely located *Pinus ponderosa* populations over 27 years. Mean environmental conditions and their spatial patterns of variation at the seed source populations were characterized based on six basic climate parameters. Despite the small area of origin, there was significant genetic variation in phenotypic plasticity for tree growth among populations. We found a significant negative correlation between phenotypic plasticity and the patch size of environmental heterogeneity at the seed source populations, but not with total environmental spatial variance. These results show that populations exposed to high microhabitat heterogeneity have evolved higher phenotypic plasticity and that the trigger was the grain rather than the total magnitude of spatial heterogeneity. Contrary to theoretical predictions, we also found a positive relationship between population plasticity and summer drought at the seed source, indicating that drought can act as a trigger of plasticity. Finally, we found a negative correlation between the quantitative genetic variance within populations and their phenotypic plasticity, suggesting compensatory adaptive mechanisms for the lack of genetic diversity. These results improve our understanding of the microevolutionary drivers of phenotypic plasticity, a critical process for resilience of long‐lived species under climate change, and support decision‐making in tree genetic improvement programs and seed transfer strategies.

## 1 INTRODUCTION

Phenotypic plasticity, that is, the ability of a genotype to express alternative phenotypes in different environments (Agrawal, 2001), is an important and widespread source of phenotypic variation that can have large effects on individual fitness (Van Buskirk et al., 1997) and population dynamics (Miner et al., 2005; Plaistow et al., 2006). Phenotypic plasticity can be adaptive if plastic responses increase fitness across environments, but can also be nonadaptive if the plastic responses decrease individual fitness (Ghalambor et al., 2007). While evolutionary responses to environmental change involve changes in allele frequencies across generations (Kinnison & Hendry, 2001; Reznick & Ghalambor, 2001), plastic responses occur without changes in the genetic structure within one generation (Sultan, 2000).

As sessile, long‐living organisms with low migration rates (Davis & Shaw, 2001), forest trees are typically exposed to a wide range of temporal and spatial environmental variation (Huey et al., 2002; Stamp & Hadfield, 2020). Moreover, trees are characterized by very long generation times such that rates of evolutionary responses can be much slower than the predicted rates of climate change (Etterson & Shaw, 2001; Savolainen et al., 2007; but see Oddou‐Muratorio & Davi, 2014). In this context, plasticity emerges as a key mechanism that allows trees for rapid responses to both environmental heterogeneity and environmental change (Valladares et al., 2014). Specifically, plastic responses may be critical to adjust to novel and/or more variable climatic conditions (Benito‐Garzon et al., 2019; Franks et al., 2014), allowing time for genetic adaptation (Chevin et al., 2010) or even directly facilitating adaptation (Ghalambor et al., 2007), and ultimately leading to increased chances of populations persistence (Nicotra et al., 2010; Richter et al., 2012).

Although the cues that trigger phenotypic plasticity are environmental, the ability to respond to those cues is genetically based and can evolve under natural selection (de Jong, 2005). Genetic variation in phenotypic plasticity (identifiable as a significant G × E interaction) is an indicator of the potential for response to selection and the evolution of plasticity (Vantienderen, 1991; Via & Lande, 1985). Over the last decades, interpretation of variation in plasticity has shifted from a troublesome source of noise in quantitative genetic studies to a fundamental characteristic that itself is under selection, evolves, and is of ecological consequence (Bradshaw, 2006; Miner et al., 2005). Tree improvement programs supply seed resources for tree plantations and have traditionally focused on reducing the effect of the G × E interaction in breeding populations. However, a better understanding of the drivers and triggers of phenotypic plasticity may provide breeders a trait for the selection of genotypes and the design of assisted migration strategies seeking to optimize tree resilience (Cooper et al., 2019).

Climate change is causing shifts in mean climatic conditions and increasing its variability (IPCC, 2019). Moreover, extreme events are increasing in frequency and magnitude (D'Odorico & Bhattachan, 2012; Jimenez et al., 2011) exacerbating selective pressures in forest trees across biomes leading to forest mortality (Hammond et al., 2022). To be able to predict how phenotypic plasticity may buffer climate change and its consequences on tree populations, we need a deeper understanding of intraspecific variation in plasticity and the environmental drivers shaping this variation (Bonamour et al., 2019). Plasticity is thought to be evolutionarily favored under specific conditions (Alpert & Simms, 2002), yet many theoretical predictions about benefits, costs, and selection on plasticity remain empirically untested (Chevin & Lande, 2011; Lind et al., 2011). One attractive study approach is to retrospectively determine the role of past environmental conditions on the evolution and differentiation of phenotypic plasticity across populations.

Theoretical models indicate that environmental heterogeneity is a major factor driving the evolution of phenotypic plasticity (Alpert & Simms, 2002; Lind & Johansson, 2007), thus influencing the divergence among plant populations in the plasticity of their traits (Matesanz et al., 2010; Weinig, 2000). Greater environmental heterogeneity is expected to select for higher phenotypic plasticity (Gianoli & Gonzalez‐Teuber, 2005; Lazaro‐Nogal et al., 2015). However, whether spatial heterogeneity selects for plasticity depends on the scale of environmental variation relative to the average dispersal distance of a species (Alpert & Simms, 2002). Habitats characterized by fine‐grained spatial heterogeneity, where environmental variation occurs at scales smaller than the dispersal distance, should favor adaptive plasticity, while coarse‐grained habitats, where environmental variation occurs at scales greater than dispersal, should favor adaptive genetic differentiation. Nevertheless, empirical studies linking the spatial scale of environmental heterogeneity and patterns of selection on phenotypic plasticity among plant populations are extremely scarce (Baythavong, 2011), especially in long‐living trees.

In spite of the ecological benefits of adaptive phenotypic plasticity, its evolution may be constrained by inherent costs (Burraco et al., 2022), and the extent of these costs is likely dependent on resource availability (DeWitt et al., 1998). Theory predicts that phenotypic plasticity should be more advantageous when mean resource availability is high rather than low (Alpert & Simms, 2002) as costs are likely to be more prominent in stressed environments (Chevin & Hoffmann, 2017; Stotz et al., 2021). When resources are more abundant, plants likely to grow faster and add and replace modules more rapidly, reducing the time required for responding to environmental cues and facilitating plants to fine‐tune plasticity to match environmental changes (Atkin et al., 2006). This hypothesis, however, has received limited empirical support and variation in plasticity across species does not appear to follow consistent patterns across resource gradients (see Stotz et al., 2021 and references therein). At the intraspecific level, few studies have assessed how phenotypic plasticity varies across tree populations adapted to distinct environments in terms of resource availability (but see Lopez‐Goldar et al., 2020; Sole‐Medina et al., 2022).

Standing genetic diversity within populations can also be related to the magnitude of phenotypic plasticity (Harter et al., 2015) and could help understand phenotypic plasticity drivers and adaptive potential of tree populations. On the one hand, high genetic diversity can promote phenotypic plasticity as it increases the chances of possessing advantageous alleles involved in the plastic responses to environmental cues (Harter et al., 2015; Hughes et al., 2008). On the other hand, phenotypic plasticity may tend to obscure selective differences among genotypes (Sultan, 1987) and thus contribute to preserve genetic variation within populations (Gomez‐Mestre & Jovani, 2013). The opposite pattern relating high phenotypic plasticity with low genetic diversity has, however, also been shown (Castillo et al., 2018; Kreyling et al., 2019). Overall, it is not yet completely clear how genotypic diversity directly relates to phenotypic plasticity and contributes to explain divergent plasticity among populations (e.g., Nicotra et al., 2010; Wortemann et al., 2011).

Genetic differentiation among populations is not only due to natural selection and adaptation to distinct environments but also depends on demographic processes (e.g., postglacial recolonization routes, bottlenecks, and founder effects) and other nonadaptive evolutionary mechanisms (e.g., genetic drift, genetic load, and mutation rates) (Lowe et al., 2017). Hence, it has been suggested that in order to detect divergent selection free of nonadaptive processes, comparisons between recently diverged populations are advantageous (Innan & Kim, 2008; Li et al., 2012). By comparing closely related populations, nonadaptive noise is removed, which provides a more reliable signal of local adaptation to more recent environmental conditions and increases our capacity to detect true environmental cues triggering selection on phenotypic plasticity. Furthermore, in order to make proper estimations of phenotypic plasticity in plant species, test environments should be within the range of environmental heterogeneity normally experienced by natural populations (Ghalambor et al., 2007). Test environments that fall far outside the range historically encountered by populations likely cause unusual stress and thus can break down genetic buffering (e.g., Rutherford, 2000, 2003) releasing cryptic genetic variation that is unexpressed under normal environmental conditions (e.g., Rutherford, 2000, 2003; Schlichting, 2004) and ultimately increasing the trait variance biasing G × E estimations.

Ponderosa pine (*Pinus ponderosa* Douglas ex Lawson) natural populations provide an outstanding model system to investigate divergent patterns of selection of phenotypic plasticity at small spatial scales. It is the most broadly distributed pine species of the Western Hemisphere (Potter et al., 2013), where it has considerable ecological and economic importance (Oliver & Ryker, 1990). Several operational taxonomic units within species have been described (Willyard et al., 2017) that may have been separated well before the last glacial maximum between 18,000 years (Potter et al., 2015) and 250,000 years ago (Lascoux et al., 2004). After the glacial retreat, varieties expanded their ranges from likely multiple refugia of unknown location, which coupled with complex mountain terrain resulted in a complex phylogeography and heterogeneous genetic structure indicating a genetic basis for climate adaptation (Johansen & Latta, 2003; Potter et al., 2013, 2015). Moreover, provenance trials of ponderosa pine have revealed abrupt genetic variation across local environmental gradients (Rehfeldt, 1990, 1991), suggesting relatively localized climate sensitivity resulting from the combined influence of local topoclimatic variation and genetically based local adaptation (McCullough et al., 2017).

To obtain a broader understanding of the microevolutionary patterns of phenotypic plasticity, we asked whether differences in climate heterogeneity and mean resource availability generate divergent selection for plasticity among closely located populations of ponderosa pine. We also tested the potential relationship between genetic diversity and phenotypic plasticity based on the differences in within‐population variability. We took advantage of three, closely located 27‐year‐old genetic trials within the geographic area of the seed source populations to calculate indexes of phenotypic plasticity for tree growth at the population level. We correlated these indexes with climate heterogeneity and resource availability at the seed source and with the estimated within‐population additive genetic variation. We addressed the following questions: (1) Do populations from more spatially heterogeneous environments show greater phenotypic plasticity? (2) Does the scale of the spatial heterogeneity contribute to explain variation in plasticity? (3) Do populations from environments with lower resource availability show more constrained phenotypic plasticity? And (4) is phenotypic plasticity related to genetic variation at the population level? We hypothesize that the magnitude of phenotypic plasticity in tree growth increases in populations from more heterogeneous and fine‐grained environments, and that populations originating from low resource environments will show limited plasticity, as well as reduced intrapopulation variation due to stronger selective pressures. Therefore, we expect a positive relationship between plasticity and genetic variation within populations.

## 2 MATERIALS AND METHODS

### 2.1 Test sites, plant material, and growth measurements

**FIGURE 1:**
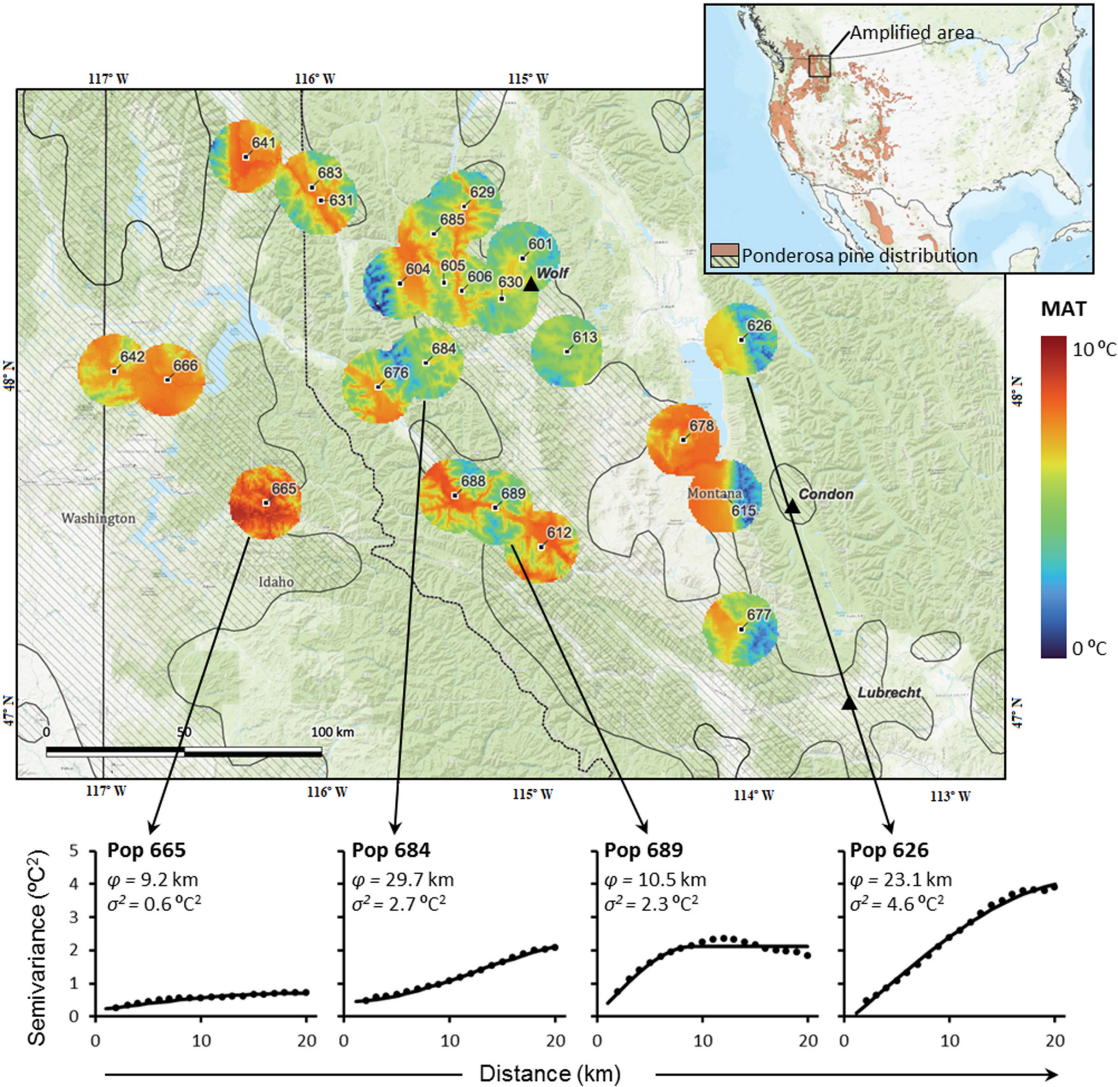
Location of the 23 studied *Pinus ponderosa* populations (black squares), planted at the three test sites (Condon, Lubrecht and Little wolf, black triangles). The upper‐right figure shows the ponderosa pine distribution (brown shaded area), which is also depicted in the main panel (striped area). For each population, the mean annual temperature (MAT, normal series 1961–1990) within a 20 km radius around the seed source is shown following a blue to red gradient. Spatial patterns of MAT within these buffers for four contrasting populations are illustrated by the corresponding semivariograms (bottom panels). The range or patch size of the spatial heterogeneity (*φ*) and the sill or total spatial variance (*σ* ^2^) are shown for these four populations (note the difference of *φ* and *σ* ^2^ parameters among the example populations).

This study was conducted on three ponderosa pine genetic trials, including 23 closely located natural populations from western Montana and Northern Idaho (see Figure 1 and Table S1). This is a genetically rich area within the species range close to the contact zone between the two widespread, traditionally defined varieties (pacific and interior), and where two different haplotypes coexist (Potter et al., 2013) and two operational taxonomic units sensu Willyard et al. (2017) overlap. The three genetic trials (Condon, Lubrecht, and Little Wolf) were planted in western Montana (USA) by the Inland Empire Tree Improvement Cooperative (IETIC; see Figure 1 and Table S2). Hence, both the studied populations and the test sites are located in a relatively small area of about 240 × 200 km within the species range.

The three common gardens followed a Population × Family structure. Seeds were collected from 115 open‐pollinated, unrelated wild mother trees in the 23 populations. Every population was represented by five open‐pollinated families, and every family was planted in all three trials. One‐year‐old bare‐root seedlings were planted in 1974 on a 3 × 3 m spacing using a randomized complete block design at the family level with four tree‐row plots and five blocks in each site. A total of 20 trees per family, 100 trees per population, and 2300 trees per test site (6900 trees in total) were established. Additional planting of containerized 2‐year‐old seedlings occurred in 1975 to replace mortality during the first year. We mapped each tree of the three trials using a submetric accuracy GPS device (Trimble Geo HX).

Tree growth was measured as tree height at ages 2, 4, 5, 11, 16, 21, and 27, and diameter at breast height (DBH; 1.4 m above ground) at ages 5, 11, 16, 21, and 27. Positive genetic correlations among ages and between height and diameter were found, and DBH at age 27 was chosen as the best predictor of genetic growth potential in these trials (de la Mata et al., 2017) and was used to estimate both population plasticity and genetic variability. Growth traits in forest trees integrate numerous physiological processes that reflect acclimation to the environment and are good fitness surrogates (Savolainen et al., 2007; Wu & Ying, 2004). Therefore, plasticity in tree growth represents the response potential of several performance‐related processes and traits (Harter et al., 2015), and when adaptive, it maintains fitness over a range of environmental conditions (Agrawal, 2001).

### 2.2 Data analysis

#### 2.2.1 Spatial adjustments in genetic trials

Since spatial autocorrelation is a common concern in forest genetic trials (Magnussen, 1990), we first corrected growth traits for spatial autocorrelation at each site independently. Spatial adjustments were made using the Iterative Spatial Analysis method (ISA; Zas, 2006) that relies on geostatistical techniques to account for spatial autocorrelation when estimating genetic parameters. Briefly, we modeled the variance of the growth traits, after adjusting for genetic effects, as a function of the distance separating the trees within each site (semivariogram). Theoretical semivariograms were then fitted to these functions and used for kriging interpolation. Kriging estimates were then used to adjust the original growth data for spatial autocorrelation. The whole process was repeated iteratively until convergence of the genetic effect estimate (Zas, 2006). All subsequent analyses were performed upon the spatially adjusted data.

#### 2.2.2 Linear mixed‐effects model

A general linear mixed model was fitted to the spatially adjusted growth data to disentangle the relative importance of the variation among families and populations, and their interaction with the environment:

(1) $$Y_{\text{ijkl}}=\mu +S_{i}+P_{j}+F{\left(P\right)}_{k\left(j\right)}+S\times P_{\mathrm{ij}}+S\times F{\left(P\right)}_{\mathrm{ik}\left(j\right)}+\varepsilon_{\text{ijkl}}$$

where *Y*_ijkl_ is the observation of the ijkl^th^ tree, *μ* is the overall mean, *S*_i_ is the fixed effect of the site *i*, *P*_j_ is the random effect of the population j, *F*(*P*)_k(j)_ is the random effect of the family k of the population j, *S* × *P*_ij_ and *S* × *F*(*P*)_ik(j)_ are the corresponding random interactions, and *ε*_ijkl_ is the random tree effect of the ijkl^th^ individual or error term. The within‐site block effect and its interactions were not included in the model since the dependent variable was already free of spatial autocorrelation. Given that the dependent variable met the assumptions of normal distribution, the mixed model was fitted using the mixed procedure of SAS (Littell et al., 2006), and variance components were estimated using the reml method. The fitted model also allowed for heterogeneity of error variances in order to account for heterogeneous standard errors of population and family means across sites. Significance of random effects was tested using log‐likelihood ratio tests comparing the restricted log‐likelihoods of the full model with respective reduced models excluding the effect to be tested (Yang, 2002). Narrow sense individual heritability (h_i_^2^) for phenotypic plasticity in growth across populations was estimated as the ratio of additive genetic variance to total phenotypic variance. The standard error of heritability was estimated by the Delta method based on asymptotic estimates of the variances and covariances of the variance components of the random model (Lynch & Walsh, 1998).

#### 2.2.3 Within‐population genetic variance

To estimate within‐population genetic variances at each site, we used the full mixed model (Equation 1), but here the population effect was considered a fixed factor. We configured a variance–covariance matrix with a separate family variance for each population including the subject and type = un(1) options in the random statement of the mixed procedure of SAS (Littell et al., 2006).

#### 2.2.4 Causes of the population by site interaction

To interpret the *S* × *P* interaction (variation in phenotypic plasticity among populations), we followed the likelihood framework proposed by Yang (2002). Under this framework, different reduced models constraining different elements of the population variance–covariance (VCOV) structure were fitted. Hypothesis testing regarding the constraints imposed on the VCOV structure was carried out by restricted likelihood ratio tests (Fry, 2004). We specifically tested for (i) heterogeneity of population variances (scale effect), (ii) deviations from perfect correlations (crossover interaction), and (iii) heterogeneity of site‐to‐site population correlations across pairs of sites (see details in Methods S1).

#### 2.2.5 Estimation of population plasticity

Plasticity estimates for every single population were obtained following the framework proposed by Denis et al. (1997), which has been also followed in other pine species for ecotype stability estimations (Voltas et al., 2018). Plasticity classical models can be readily embedded in a mixed model framework (Denis et al., 1997; Piepho, 1999) in which population acts as a fixed effect, and site and population by site interaction act as random effects. Mixed modeling allows to model the (S × P)_ij_ term in a flexible way (Denis et al., 1997), and classical stability approaches for describing such effect were handled using appropriate variance–covariance (VCOV) structures (see details in Methods S2). We fitted an additive mixed‐effects model in which populations do not differ in stability, and four different stability models to accommodate among‐population variation in stability: Shukla's stability model, Finlay–Wilkinson regression, Eberhart–Russell regression and the additive main effect and multiplicative interaction model AMMI‐1 (see complete explanations in Methods S2). The superiority of different VCOV structure‐based models was compared by information criteria such as Akaike's information criterion (AIC) and Bayesian information criterion (BIC). Both statistics are in the smaller‐is‐better form.

#### 2.2.6 Characterization of climate at the seed source locations

Climate at the seed source locations was characterized based on six basic parameters with relevant effects on biological processes and on broader data availability: mean annual temperature (*MAT*, °C), mean temperature of the warmest month (*MTWM*, °C), mean temperature of the coldest month (*MTCM*, °C), annual precipitation (*MAP*, mm), summer precipitation (*MSP*, May to September, mm), and summer heat‐moisture index [*SHMI* = (*MTWM*)/(*MSP*/1000)]. These variables were selected based on results from prior studies examining growth responses to climate in ponderosa pine (McCullough et al., 2017; Rehfeldt et al., 2014). Climate data for the normal series (1961–1990 period) were generated for every seed source location on a 1 × 1 km grid within a radius of 20 km from the center of the seed source (considering this wide area as relevant for the evolutionary trajectory of the populations) with the ClimateNA v5.21 software package (Wang et al., 2016) (Table S1, Figure 1). This software locally downscales historical climate data layers into scale‐free point estimates of climate values. The 1 × 1 km grid point locations were obtained using QGis 3.24.1. Mean data across the normal period within the 20 km radius for each parameter were used as proxies of the mean climate conditions for each population (Table S1). The normal period 1961–1990 used to characterize the evolutionary environment of the studied populations offers several advantages including good climate‐station coverage, conditions are the onset of a major anthropogenic warming signal and a period of global dimming in the 1950s to 1980s may have masked a small anthropogenic warming signal (Marchi et al., 2020).

#### 2.2.7 Climate heterogeneity at the seed source locations

Climate heterogeneity at each seed source location was estimated using geostatistical approaches. Semivariograms were constructed for the six climatic parameters described before considering the 20 km radius from the center of each population (see details in Methods S3). Spherical, Exponential, or Gaussian models were fitted to the empirical semiovariograms using nonlinear regression procedures, and the best model selected based on the goodness of fit (adjusted nonlinear R^*2*^). The *sill* or *max variance* and the *patch size* of the spatial patterns were then extracted from the theoretical semivariogram models, and used as descriptors of the intensity and scale of climate heterogeneity, respectively.

#### 2.2.8 Effects of climate heterogeneity, resource availability, and genetic variation on population phenotypic plasticity

We tested whether greater climate heterogeneity and increased resource availability selected for higher magnitudes of phenotypic plasticity. We performed Pearson's correlations among phenotypic plasticity for each population across the three test environments and the climate heterogeneity and means of climate parameters at the origin of each seed source. We also correlated population plasticity with the within‐population additive genetic variation.

## 3 RESULTS

### 3.1 Variance components for the full mixed model

**FIGURE 2:**
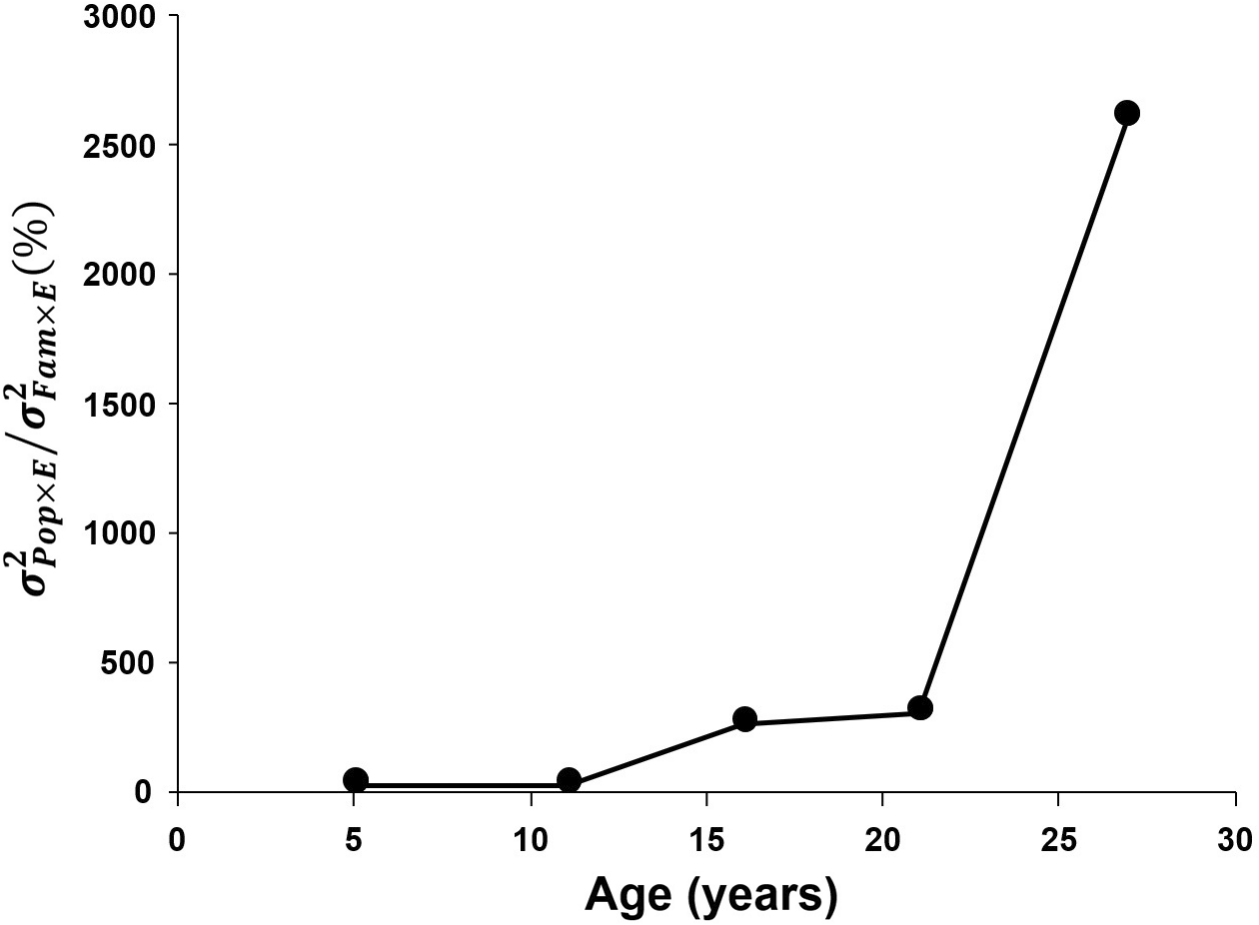
Relative relevance of the population by site interaction variance (*σ* ^2^_Pop × Site_) versus the family by site interaction variance (*σ* ^2^_Fam × Site_) for diameter growth over time from a joint analysis including the three test sites. The relative contribution of both sources of genetic variation is expressed as: (*σ*_Pop × Site_^2^ */σ*_Fam × Site_^2^) × 100.

Results from the full mixed model (Table 1, Equation 1) showed a highly significant site effect on growth (*p* < 0.001), evidencing significant plastic responses to the environmental conditions at each test site. Growth was highest at Condon (11.6 m and 18.7 cm in average tree height and DBH at age 27, respectively), intermediate at Little Wolf (9.1 m in height and 17.8 cm in DBH), and lowest at Lubrecht (7.5 m in tree height and 15.5 cm in DBH) (Figure S3). Family within‐Population effect accounted for the largest amount of variation (Table 1, *p* < 0.001), while the Population effect was not significant. Thus, most of the genetic variation in tree growth occurred within populations. By contrast, Family × Site interaction was not significant while Population × Site was (*p* < 0.01), showing that genetic variation in plasticity was Population driven. Population × Site interaction variance for DBH increased with tree age, and it was up to 28‐fold larger than Family × Site interaction variance at age 27 (Figure 2). The narrow sense heritability estimate for phenotypic plasticity across populations was statistically significant (0.06 ± 0.03).

**TABLE 1:** Summary of the mixed model analysis for diameter at breast height assessed at age 27 across sites (Equation 1). For the site fixed effect, *F*‐ratio and associated significance level is shown; for random effects, variance component estimates ± standard errors (VC ± SE) and associated significance levels of the log‐likelihood ratio tests^a^ for significance of each variance component are shown.

Source of variation	*F*_SITE_ ^b^		VC ± SE		VC (%)
Fixed effects					
Site	98.74	***			
Random effects					
Population			0.144 ± 0.174	ns	0.4
Family (Population)			0.911 ± 0.219	***	4.3
Site × Population			0.329 ± 0.140	**	0.6
Site × Family (Population)			0.012 ± 0.157	ns	0.1
Error Condon			15.935 ± 0.607		
Error Lubrecht			18.510 ± 0.725		
Error Little Wolf			24.775 ± 0.948		

^a^ Asterisks denote the significance level (***p < 0.001; **p < 0.01; *p < 0.05; ns = *p* > 0.05) associated to the chi‐square value given by the difference in two times the log likelihood of that factor included versus excluded from the model. Because variance components are constrained to be positive, test of variance components are one‐tailed (Fry, 2004).

^b^ *F*_2,45.6_.

### 3.2 Causes of the population by site interaction

A comprehensive likelihood‐based analysis regarding the relevance and interpretation of the Population × Site interaction is summarized in Table 2. Results show that the lack of perfect population correlation between sites, and not the heterogeneity of population variance across sites, is the main cause of the interaction (the hypothesis of homogeneous population variances cannot be rejected, Table 2). Hence, there is a crossover interaction responsible for changes in population rankings, which is the biologically important source of the G × E interaction. Moreover, results indicate that the population covariance across sites was not constant (Table 2). Population growth correlations between Condon and Lubrecht, Condon and Little Wolf, and Lubrecht and Little Wolf were 0.02 ± 0.34 (*p* > 0.05), 0.62 ± 0.24 (*p* < 0.05) and 0.87 ± 0.21 (*p* < 0.01), respectively, indicating that Lubrecht and Condon are the more dissimilar sites in terms of population rankings.

**TABLE 2:** Likelihood ratios for testing different hypotheses on the relevance and interpretation of the population by environment interaction for diameter growth at age 27.

Null hypotheses	df	*χ* ^2^	*p* > *χ* ^2^
Homogeneity of population variance across sites	2	1.44	0.486
Perfect population correlation between all site pairs	3	16.20	<0.01
Homogeneity of population covariance across site pairs	2	7.53	<0.05

*Note*: The chi‐squared values shown are the differences in two times the log‐likelihood of the full model (unstructured variance–covariance [VCOV] matrix) and different reduced models constraining different elements of the VCOV structure across sites (see Methods S1). Degrees of freedom (df) associated with the chi‐squared values result from the difference between the number of VCOV parameters specifying the full and reduced models. *p*‐Values lower than 0.05 indicate that the null hypotheses should be rejected.

### 3.3 Estimation of population plasticity indexes

The best fitting model for population stabilities of tree DBH at age 27 based on the lowest AIC and BIC values favored the Eberhart–Russell model (Table 3). Hence, plasticity (opposite of stability) in each population was estimated based on this model and was used for subsequent correlations with climate heterogeneity, resource availability, and genetic variation in each population. According to this model, variation in plasticity among populations was large, with stability estimates ranging between 0.55 and 1.40 (2.5‐fold range).

**TABLE 3:** Goodness‐of‐fit statistics of several stability mixed models to accommodate the among‐population variation in plasticity for tree diameter at age 27.

Model	Number parms^a^	Goodness of fit statistics^b^	
		AIC	BIC
Additive	3	147.7	145.9
Finlay–Wilkinson	24	154.0	132.4
Shukla	25	157.7	136.1
AMMI‐1	25	155.9	133.3
Eberhart–Russell	46	**144.9**	**104.3**

^a^ Number of random parameters.

^b^ AIC, Akaike's information criterion; BIC, Bayesian information criterion. All statistics is in smaller‐is‐better form; bolded values indicate preferred models according to either AIC or BIC criterion and thus the model choice.

### 3.4 Climate heterogeneity at the seed source locations

All climate parameters for all seed source locations revealed nonrandom spatial structures. Hence, for all populations and within a 20 km radius from the center, climate data from closer locations were more similar than those from farther locations. The spherical theoretical semivariogram fitted well to the observed semivariogram in 80 of 138 seed source location by climate parameter combinations, while Gaussian semivariograms fitted better in 51 cases and exponential semivariograms in seven cases (Table S4 in Methods S3). These results indicate widespread patchy spatial structures for environmental heterogeneity, given that both spherical and Gaussian semivariograms show a clear asymptotic *sill* (maximum variance) at which the data cease to be correlated which determines the patch size of spatial heterogeneity. Patch sizes were widely variable among populations and climate parameters ranging from 8 to 112 km (coefficients of variation across populations varying from 0.37 [for MAT and SHM] to 1.03 [for MSP]) (Table 4), pointing to different spatial scales of climate heterogeneity depending on the seed source location. Furthermore, the maximum variance among patches showed even larger coefficients of variation across populations than patch size (ranging from 0.75 for SHM to 1.59 for MTCM) (Table S5 in Methods S3), which suggests distinct magnitudes of total variation among patches of heterogeneity depending on the seed source.

**TABLE 4:** Patch size (range, *φ*) of environmental heterogeneity in km derived from the theoretical fitted semivariograms for every climate parameter in every population.

Population	MAT^a^	MTWM^b^	MTCM^c^	MAP^d^	MSP^e^	SHM^f^
601	20.6	20.7	22.2	19.3	112.4	23.3
604	22.4	24.0	20.4	26.1	26.6	21.2
605	8.9	8.7	11.1	9.7	9.4	9.8
606	9.3	9.2	11.4	18.4	13.4	9.1
612	14.6	15.6	15.5	17.4	15.3	12.2
613	16.3	45.1	42.4	30.7	37.5	20.7
615	28.6	27.9	30.3	27.7	28.3	29.5
626	23.1	21.2	21.3	16.3	15.2	18.6
629	15.5	15.4	16.6	15.1	14.3	15.0
630	10.5	11.0	10.7	63.7	13.1	13.1
631	14.2	13.7	12.3	12.2	13.0	11.8
641	17.2	19.3	13.3	14.2	14.2	11.9
642	9.4	9.7	16.2	10.9	10.7	9.5
665	9.2	10.9	10.9	17.0	16.6	13.8
666	13.3	12.8	35.7	11.9	11.8	12.2
676	22.8	24.6	29.7	17.2	17.5	14.3
677	18.5	17.7	19.9	20.3	21.5	18.8
678	12.1	9.9	50.3	9.3	10.6	11.1
683	14.2	13.9	12.6	10.9	10.9	11.3
684	29.7	31.8	18.7	27.5	26.1	25.9
685	19.0	18.2	17.5	16.9	14.2	16.3
688	14.6	13.9	13.5	11.8	12.7	11.9
689	10.5	9.8	12.9	13.5	8.8	9.7
Coefficient of variation across populations	0.37	0.50	0.53	0.60	1.03	0.37

*Note*: Empirical semivariograms were constructed upon climate data based on means of the normal series period (1961–1990) at every location in a 1 × 1 km grid within a radius of 20 km from the center of each population source location.

^a^ MAT: mean annual temperature (°C).

^b^ MTWM: mean temperature of the warmest month (°C).

^c^ MTCM: mean temperature of the coldest month (°C).

^d^ MAP: mean annual precipitation (mm).

^e^ MSP: mean annual summer (May to September) precipitation (mm).

^f^ SHM: summer heat‐moisture index ([MTWM]/[MSP/1000]).

### 3.5 Effects of climate heterogeneity, resource availability, and genetic variation on population phenotypic plasticity

**FIGURE 3:**
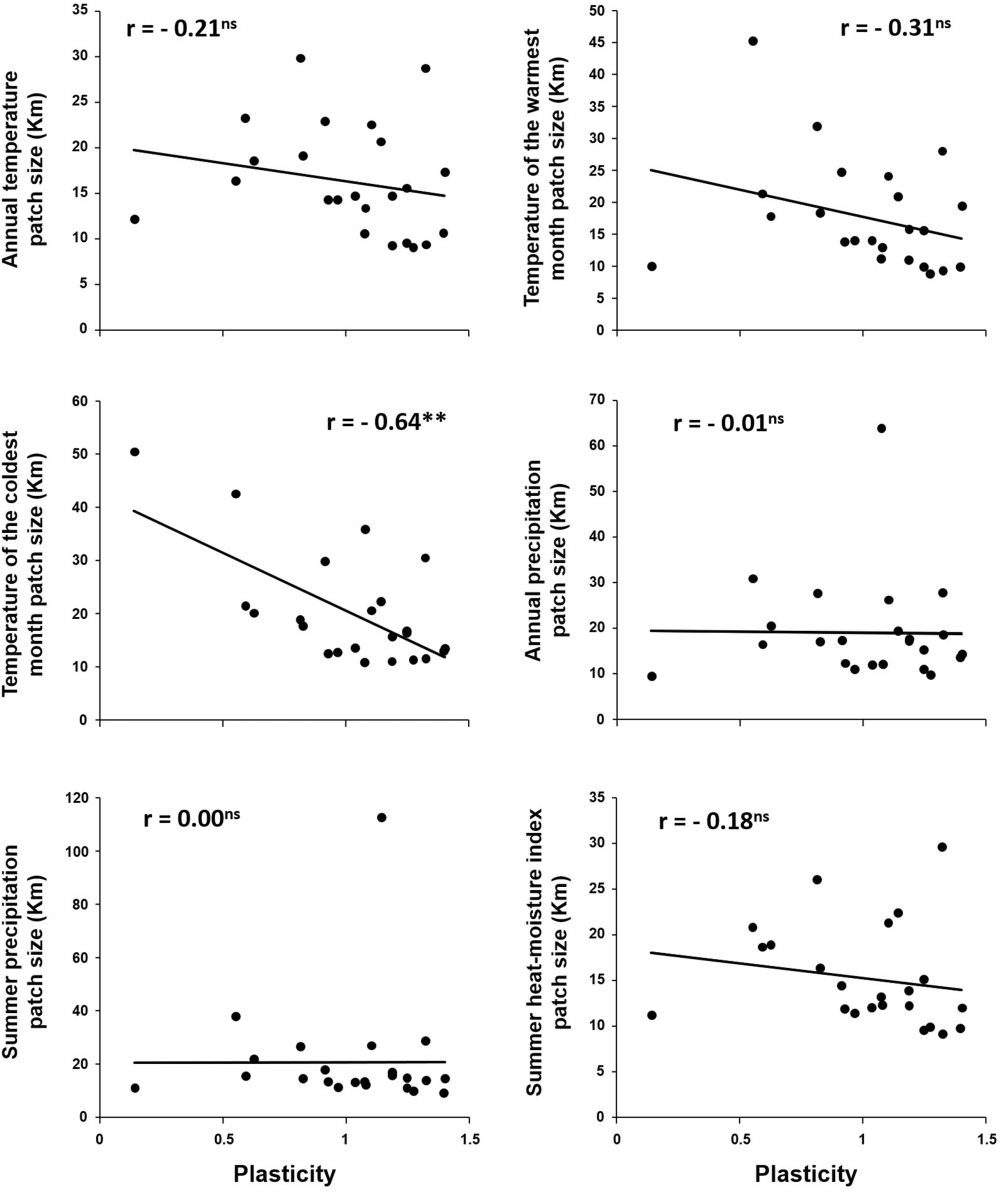
Patch size of environmental heterogeneity as predictor of the phenotypic plasticity in growth of 27 ponderosa pine populations planted across three test sites. The figures show the correlations between the patch size of environmental heterogeneity for main climate parameters (MAT, MTWM, MTCM, MAP, MSP, and SHM, see Table 4 for variable description) at the site of origin and the phenotypic plasticity in diameter estimated through the Eberhart–Russell stability model (Eberhart & Russell, 1966). For each population, the patch size (range, *φ*, in km) was estimated fitting a theoretical semivariogram to the climate dataset upon the means of the normal series period (1961–1990) at every 1 × 1 km grid within a radius of 20 km from the center of the seed source. Regression lines and Pearson *r* coefficients are shown. Asterisks indicate a significant *p*‐value < 0.05. Dots depict populations (*n* = 23 populations).

We did not find a correlation between plasticity and the maximum variance (*sill*) of the spatial pattern of heterogeneity at the seed source of each population. However, we found that correlations between plasticity and patch size at the seed source were generally negative (Figure 3), and statistically significant for the mean temperature of the coldest month (*r* = −0.64; *p* < 0.01). These results suggest that more plastic populations originated in more heterogenous environments (smaller patch sizes) in terms of temperature of the coldest month.

**FIGURE 4:**
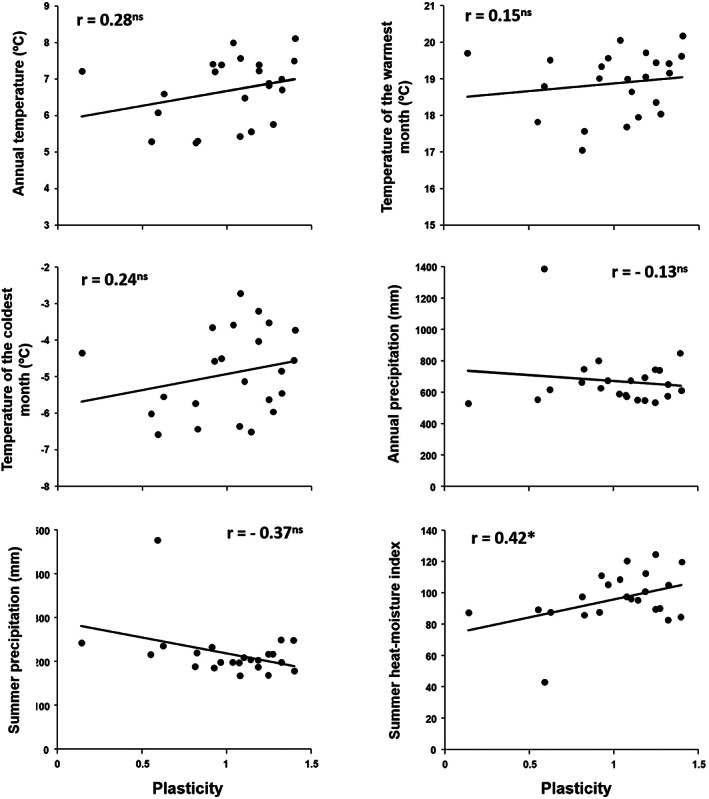
Mean resource availability as predictor of the phenotypic plasticity in growth of 27 ponderosa pine populations planted across three test sites. The figures show the correlations between the main climate parameters at the site of origin (MAT, MTWM, MTCM, MAP, MSP, and SHM, see Table 4 for variable description) and the phenotypic plasticity in diameter estimated through the Eberhart–Russell stability model (Eberhart & Russell, 1966). Climate parameters were estimated as the means over the normal series period (1961–1990) at every population source location. Regression lines and Pearson *r* coefficients are shown. Asterisks indicate a significant *p*‐value < 0.05. Dots depict populations (*n* = 23 populations).

Correlations between plasticity and the mean value of climate parameters for each population showed a general trend of greater plasticity in hotter and drier sites, with statistically significant results for summer heat‐moisture index (*r* = 0.42; *p* = 0.044) (Figure 4). Thus, more plastic populations belong to environments with stronger summer drought.

**FIGURE 5:**
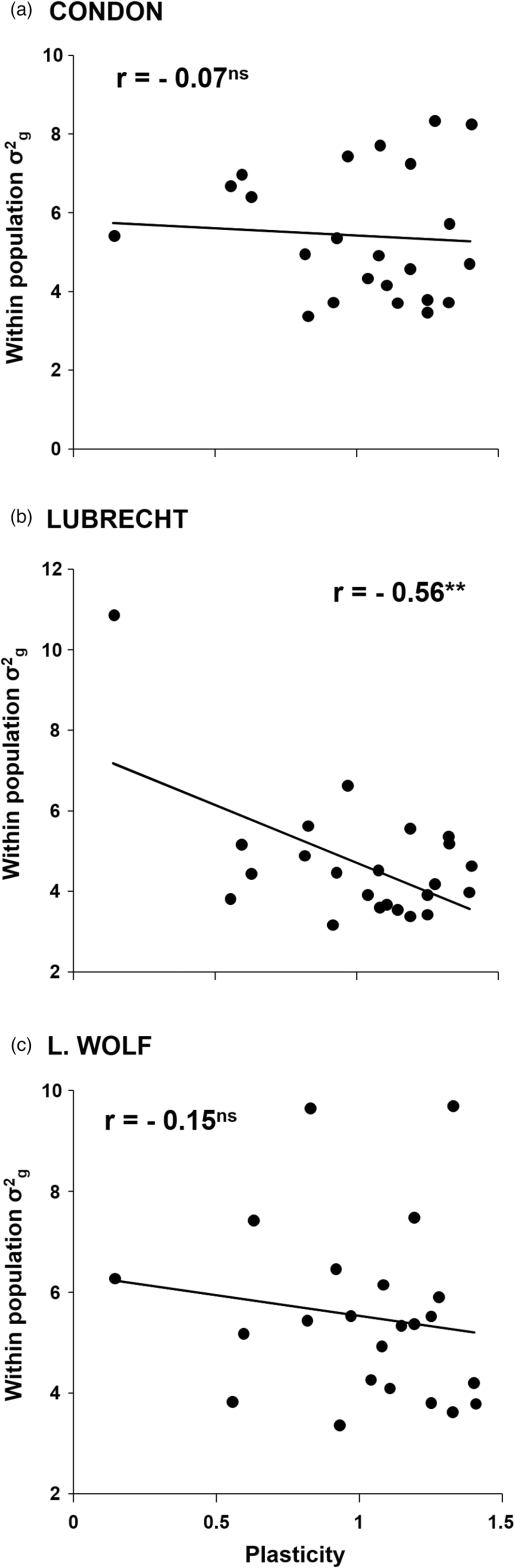
Correlations between population phenotypic plasticity across the three test sites and within‐population family variance estimated at each test site Condon (a), Lubrecht (b) and Little wolf (c) separately. Regression lines and Pearson *r* coefficients are shown. Asterisks indicate significant *P*‐values (** <0.01). Dots depict populations (*n* = 23 populations).

Correlations between phenotypic plasticity and within‐population genetic variation for each population showed a negative general trend at the three test sites (Figure 5) but were only significant at Lubrecht (*r* = 0.56; *p* = 0.005), the site with more constrained growth.

## 4 DISCUSSION

Under a scenario of climate change, phenotypic plasticity is critical for the resilience of populations of long‐lived species where long generation times limit rapid evolutionary change. Here, we provide evidence for genetic variation in the magnitude of plastic responses to environmental variability in closely located populations of a forest tree species. We also show some evidence that fine‐grained spatial heterogeneity and resource availability are related to phenotypic plasticity in tree growth. Overall, our results show differentiation in phenotypic plasticity among plant genotypes. This differentiation was mostly population driven, such that genotypes from the same population showed similar plastic responses across the three test sites but different from other populations suggesting that plastic responses are contingent upon the environment where populations evolved. Our results also provide support to the long‐hypothesized role of environmental heterogeneity on phenotypic plasticity. Populations that have been exposed to higher microhabitat heterogeneity likely through recent evolutionary time showed higher phenotypic plasticity and thus may be better equipped to respond to changing climates than populations from more homogenous environments. Importantly, we found that the patch size of environmental heterogeneity but not the total variance among patches was the driver of phenotypic plasticity, suggesting that the grain of spatial heterogeneity rather than the total magnitude drive evolution of plasticity in highly heterogeneous forest ecosystems. Moreover, trees from populations on drier sites were more plastic than trees from populations on mesic sites, providing evidence that reduced water availability, a common stress, triggers the evolution of phenotypic plasticity. Finally, phenotypic plasticity was negatively linked to quantitative genetic variation in tree growth, suggesting compensatory mechanisms of adaptation for the lack of genetic diversity and that phenotypic plasticity does not prevent depletion of genetic variance due to natural selection. Overall, our study highlights the need to consider within species population differentiation in phenotypic plasticity in order to provide insights into species' range shifts under climate change and into seed transfer strategies in assisted migration programs.

### 4.1 Phenotypic plasticity driven at the population level: Cue of local adaptation

Tree growth depends on resource availability and strongly influences individual success among competing individuals, mainly during early seedling establishment when density‐dependent mortality in trees typically occurs (Dodd & Silvertown, 2000) and when the proportion of seeds which attain maturity is usually very small (Petit & Hampe, 2006). Phenotypic plasticity of tree growth is likely to be beneficial when environmental conditions are more variable, because of changing costs and benefits of investing in growth versus other functions as a function of environmental conditions (Harter et al., 2015). In this study, the significant population by environment interaction but not family within population by environment interaction provides evidence of homogenous within‐population plastic responses but dissimilar patterns of plasticity among populations. Hence, despite the spatial proximity of the studied populations and their likely substantial genetic relatedness, each population appears to have a unique genetic pattern where plasticity plays a different role for coping with environmental change (Gibert et al., 2004). These results are consistent with results showing that local adaptation in forest tree species is often detected despite high gene flow (Savolainen et al., 2007) and, as in the case for ponderosa pine in our study region (Rehfeldt, 1991), it occurs at small spatial scales (Eckert et al., 2015 and references therein). Divergent phenotypic plasticity among populations has been also found in other pine species (Vizcaino‐Palomar et al., 2020; Voltas et al., 2018).

Moreover, the significant population by environment interaction was due to departures from perfect population correlations between environments, pointing to crossover interaction effects (Yang, 2007). Crossover interactions are those biologically relevant as they involve rank changes in tree performance and, consequently, different signs of plastic responses when subjected to varying environmental conditions. The unevenly distributed plasticity among ponderosa pine populations we find here may be related to the historical environmental conditions at the seed source and could potentially lead to differential short‐term adaptability to climate variability. Indeed, prior studies at regional scales revealed geographic patterns of genetic variation among populations of *P. ponderosa* along elevation gradients, a surrogate for temperature (Rehfeldt et al., 2014 and references therein; Sorensen et al., 2001). These patterns are consistent with natural selection (Morgenstern, 1996) and are interpreted as responses to selection along temperature gradients. The complex mountain terrain (Madsen & Blake, 1977) combined with the high genetic diversity of ponderosa pine across our study area (Potter et al., 2013; Willyard et al., 2017) have likely contributed to divergent selection and local adaptation for phenotypic plasticity.

### 4.2 Grain but not magnitude of spatial heterogeneity drives evolution of phenotypic plasticity

Our results show that the degree of phenotypic plasticity in growth of ponderosa pine populations was positively correlated with climate heterogeneity at the seed source locations. This is consistent with empirical (Gianoli, 2004; Gianoli & Gonzalez‐Teuber, 2005; van Kleunen & Fischer, 2005) and theoretical studies (Alpert & Simms, 2002; Bradshaw & Hardwick, 1989; Matesanz et al., 2010) showing that phenotypic plasticity can be related to the environmental heterogeneity a population is subjected to, including conifer (Vizcaino‐Palomar et al., 2020) and broadleaved (Balaguer et al., 2001) forest tree species. However, most of these previous studies focused on temporal, as opposed to spatial, variability as the source of heterogeneity, despite the fact that spatial variability adds environmental complexity (Scheiner, 2013), and in some cases, it has been shown to affect plasticity (Ernande & Dieckmann, 2004; Sultan & Spencer, 2002). This study contributes to the rare evidence that spatial heterogeneity facilitates phenotypic plasticity (see Lind & Johansson, 2007). Hence, populations exposed to stronger spatial climate heterogeneity develop greater plasticity in tree growth likely retaining high fitness for a wider range of environments relative to populations from more spatially homogeneous locations (De Kort et al., 2020).

Importantly, ponderosa pine populations from environments characterized by smaller patch sizes of climate heterogeneity (patchier or fine‐grained environments) were the ones showing higher magnitudes of plasticity, while total variation among patches was not related to plasticity. Therefore, we also provide rare evidence that the grain of spatial heterogeneity rather than the total magnitude can be a more relevant trigger of phenotypic plasticity. In fine‐grained environments, the progeny has more chances to experience a range of environmental variation within the dispersal breath of the mother tree, and selection should favor adaptive phenotypic plasticity. Adaptive plasticity enables dispersing seedlings to maximize fitness by appropriately matching their phenotype to the environment where they are established. By contrast, in coarse‐grained environments, dispersed seeds are more likely to experience a homogeneous environment similar to that of their mother tree, and selection for plasticity should be relatively weaker or should favor canalized trait expression if costs of plasticity are sufficiently strong (Baythavong, 2011; Sultan & Spencer, 2002).

Our results showing that heterogeneity in mean temperatures of the coldest month contributes to phenotypic plasticity of growth are consistent with prior studies reporting that winter cold temperatures or proxy variables are the best predictors of growth potential in *P. ponderosa* populations (Sorensen et al., 2001). Strong relationships between population elevation and genetic variation provide additional indirect support of the strong effect of cold temperatures (e.g., Rehfeldt et al., 2014). However, although cold winter temperatures influence ponderosa pine growth potential and genetic variability, it is important to note that low temperature control of growth can be decoupled from that of growth plasticity (Kusmec et al., 2017). Indeed, the broadly supported model, particularly for tree growth (Wu, 1998), to explain the genetic basis for phenotypic plasticity, the epistasis model (or gene regulation model), poses that phenotypic plasticity is due to the interaction of genes that determine the magnitude of responses to environmental effects with those that determine the average expression of the trait (van Heerwaarden & Sgrò, 2017).

### 4.3 Reduced water availability selects for increased phenotypic plasticity

Consistent with some studies (see below), our results show greater plasticity in populations from dryer and hotter environments. However, the degree to which resource availability enhances or prevents phenotypic plasticity is highly controversial. On the one hand, theory predicts phenotypic plasticity to be more advantageous under high resource availability (Alpert & Simms, 2002), and a number of studies support this prediction (Stotz et al., 2021; Valladares et al., 2007), including phenotypic plasticity for growth (Santos‐del‐Blanco et al., 2013) and photosynthetic traits (Baquedano et al., 2008) in a Mediterranean pine species. Consistently, adaptation to harsh environments is usually associated with phenotypic stability to maintain a conservative resource‐use strategy (Sole‐Medina et al., 2022; Voltas et al., 2018). On the other hand, several studies have documented higher plasticity in plant populations from more stressful environments (Kreyling et al., 2019; Lazaro‐Nogal et al., 2015), including tree species (Cooper et al., 2019). An explanation for this discrepancy is that populations from environments where stress is unusual have not evolved the capacity to exhibit flexible plastic responses to tackle reductions in resource availability (Gianoli & Gonzalez‐Teuber, 2005). Throughout most of its distribution, ponderosa pine is subjected to summer drought. Prior work in ponderosa pine seedlings showed that those from the most drought tolerant population were more responsive to water availability than those from other populations, such that they used water quickly when water was available, but closed their stomata in response to water stress (Zhang et al., 1997). This suggests that, in populations from dryer sites, a consistently conservative water use strategy (low plasticity) could represent a large opportunity cost when water is available. Consistent with this plastic response, we show that populations from locations where summer drought is more intense exhibit higher phenotypic plasticity, pointing to reduced resource availability as a trigger of plastic responses.

### 4.4 Increased phenotypic plasticity is related to lower within‐population genetic variation

Our results show that populations with higher phenotypic plasticity showed lower quantitative genetic variation within populations. These results are in line with prior studies in other plant species (Castillo et al., 2018; Kreyling et al., 2019) including the Mediterranean Stone pine (*Pinus pinea* L.; Vizcaino‐Palomar et al., 2020). This pattern has been interpreted as a compensatory mechanism for the lack of genetic diversity to cope with environmental change. However, the literature is not clear on the degree to which within‐population genetic diversity is associated with the magnitude of phenotypic plasticity (e.g., Nicotra et al., 2010; Wortemann et al., 2011). Some other studies suggest that genetic diversity facilitates phenotypic plasticity (Harter et al., 2015; Jump et al., 2009) because genetic variation within populations may increase the chances of possessing allele combinations that are advantageous for responding to environmental variation (Jump et al., 2009; Nicotra et al., 2010). Theoretical models predict that plasticity can slow adaptive evolution in response to environmental variation by obscuring selective differences among genotypes and maintaining genetic variation largely unavailable to selection (Chevin et al., 2010). Alternatively, plasticity can enhance evolutionary responses in novel or fluctuating environments by allowing populations persistence, thus giving selection time to work (Lande, 2014). Hence, plastic responses can facilitate selection to effectively work what may bring as consequence a depletion of genetic variability as we find here.

## 5 CONCLUSIONS AND APPLICATIONS

We provide empirical evidence of among‐population variation in the magnitude of plastic responses to environmental heterogeneity in a forest tree species based on a 27‐year experiment. In addition, we found positive relationships between fine‐grained climate heterogeneity and reduced water availability at the site of population origin and the magnitude of phenotypic plasticity. These results improve our understanding of the microevolutionary drivers of phenotypic plasticity, a trait that is critical for the resilience of long‐lived species under climate change, where long generation times limit rapid evolutionary change.

In spite of the fundamental role that phenotypic plasticity plays in the resilience of tree populations, most approaches for species distribution models have traditionally considered species as uniform units without accounting for phenotypic plasticity and differences in phenotypic plasticity among populations (Parmesan, 2006; discussed in Valladares et al., 2014). Our study highlights the need to consider phenotypic plasticity variation within species even at local spatial scales in order to provide insights into species' range shifts under climate change.

Overall, our results suggest that plasticity may constitute an important tool for forest managers, particularly within tree breeding and tree genetic conservation programs. Tree improvement programs supply seed resources for managed tree plantations, and for restoration purposes after natural and human‐caused disturbances (e.g., fire, severe drought, and reclamation). These programs have traditionally considered genetic variation in plasticity as a troublesome source of noise which hinders selection of genotypes fitted to a wide range of environments. Based on our results, populations exhibiting higher plasticity can be expected to outperform less plastic populations in more variable environments (Harter et al., 2015; Nicotra et al., 2010; Richter et al., 2012). Hence, the magnitude of phenotypic plasticity for genotypes and populations should be a trait under consideration in selection programs when seeking to optimize tree resilience under warming and drying climate (Cooper et al., 2019).

Our study also has implications for assisted migration programs and the estimation of transfer functions to assess the suitability of a given population to a given location. Transfer functions are estimated based on the climate at the seed origin and at the planting location (O'Neill et al., 2014), and indirectly incorporate phenotypic plasticity informed from multi‐environmental provenance trials to estimate transfer distances. However, transfer functions do not account for the environmental heterogeneity at the new environment. Based on our results, environmental heterogeneity both at the seed source and at the new location should be taken into account in assisted migration guidelines to match transferred populations to their new environments.

Finally, our study serves to guide future research on environmental triggers of phenotypic plasticity. Additional studies, across the range of ponderosa pine and other species, are required to corroborate the environmental triggers of local variation in phenotypic plasticity. To that end, increasing the number of test sites across local environments would improve the accuracy of population plasticity estimations. Furthermore, using alternative parameters beyond climate (e.g., soil type and slope) as descriptors of local environments would increase the ability to detect environmental cues triggering phenotypic plasticity. Moreover, growth is a complex process and additional fitness related traits (e.g., physiological traits) should be studied to gain a deeper understanding of adaptive plastic responses under environmental change.

## CONFLICT OF INTEREST

The authors declare that there is no conflict of interest.

## Supporting information


Appendix S1

## Data Availability

Raw data were submitted to DIGITAL.CSIC repository, and accession numbers will be supplied upon request.
