# Supplementary material for: Drivers of population differentiation in phenotypic plasticity in a temperate conifer: A 27‐year study
Source: Evol Appl. 2022 Oct 31;15(11):1945–62. doi: 10.1111/eva.13492 (PMC9679231; doi:10.1111/eva.13492)

**SUPPORTING INFORMATION**

**Drivers of population differentiation in phenotypic plasticity in a temperate conifer: a 27-year study**

Raúl de la Mata, Rafael Zas, Gloria Bustingorri, Luis Sampedro, Marc Rust, Ana Hernández-Serrano, Anna Sala

**Table SI.1. Geographic location, elevation and mean climate of the 23 pine populations.**

Table SI.1 Location of seed sources and their main geographic and climatic factors (normal series 1961-1990 period) for the 23 populations planted across the three test sites.

| **Population** | **Latitude (º)** | **Longitude (º)** | **Elevation**  **(m)** | **MAT**  **(ºC)^1^** | **MWMT**  **(ºC)^2^** | **MCMT**  **(ºC)^3^** | **MAP**  **(mm)^4^** | **MSP**  **(mm)^5^** | **SHM^6^** |
| --- | --- | --- | --- | --- | --- | --- | --- | --- | --- |
| 601 | 48.3779 | 114.9876 | 1036 | 5.5 | 17.2 | -6.2 | 548 | 202 | 85.1 |
| 604 | 48.3054 | 115.5905 | 914 | 6.5 | 17.9 | -4.9 | 672 | 208 | 86.3 |
| 605 | 48.3054 | 115.3752 | 1164 | 5.7 | 17.3 | -5.7 | 736 | 215 | 80.5 |
| 606 | 48.2763 | 115.2891 | 914 | 6.7 | 18.4 | -5.2 | 648 | 196 | 93.8 |
| 612 | 47.4335 | 114.9343 | 914 | 7.4 | 19.0 | -3.8 | 544 | 185 | 102.5 |
| 613 | 48.0695 | 114.7830 | 1158 | 5.3 | 17.1 | -5.6 | 551 | 215 | 79.7 |
| 615 | 47.5793 | 114.0382 | 1006 | 7.0 | 18.7 | -4.5 | 573 | 248 | 75.4 |
| 626 | 48.0880 | 113.9321 | 1097 | 6.1 | 18.2 | -6.2 | 1384 | 475 | 38.3 |
| 629 | 48.5521 | 115.2675 | 792 | 6.9 | 18.7 | -5.4 | 530 | 167 | 112.1 |
| 630 | 48.2473 | 115.0953 | 1097 | 5.4 | 17.0 | -6.1 | 580 | 195 | 87.2 |
| 631 | 48.5810 | 115.9715 | 732 | 7.2 | 18.6 | -4.3 | 623 | 184 | 101.2 |
| 641 | 48.7249 | 116.3391 | 549 | 8.1 | 19.5 | -3.5 | 609 | 177 | 110.1 |
| 642 | 48.0270 | 116.9890 | 792 | 6.8 | 17.7 | -3.2 | 741 | 215 | 82.4 |
| 665 | 47.5934 | 116.2570 | 1067 | 7.2 | 18.3 | -3.0 | 690 | 201 | 90.9 |
| 666 | 47.9980 | 116.7306 | 690 | 7.6 | 18.3 | -2.6 | 570 | 166 | 110.2 |
| 676 | 47.9673 | 115.7063 | 671 | 7.4 | 18.3 | -3.4 | 798 | 231 | 79.3 |
| 677 | 47.1438 | 113.9881 | 1067 | 6.6 | 18.8 | -5.3 | 614 | 234 | 80.5 |
| 678 | 47.7679 | 114.2307 | 1097 | 7.2 | 19.0 | -4.0 | 527 | 241 | 79.0 |
| 683 | 48.6241 | 116.0153 | 732 | 7.4 | 18.8 | -4.3 | 671 | 196 | 95.8 |
| 684 | 48.0436 | 115.4713 | 1067 | 5.2 | 16.3 | -5.5 | 660 | 186 | 87.4 |
| 685 | 48.4650 | 115.4183 | 1097 | 5.3 | 16.8 | -6.2 | 745 | 218 | 76.9 |
| 688 | 47.6084 | 115.3427 | 792 | 8.0 | 19.3 | -3.4 | 585 | 196 | 98.4 |
| 689 | 47.5648 | 115.1503 | 762 | 7.5 | 19.0 | -4.4 | 846 | 247 | 77.1 |

^1^ MAT: mean annual temperature (°C)

^2^ MTWM: mean temperature of the warmest month (°C)

^3^ MTCM: mean temperature of the coldest month (°C)

^4^ MAP: mean annual precipitation (mm)

^5^ MSP: mean annual summer (May to Sept.) precipitation (mm)

^6^ SHM: summer heat-moisture index ((MTWM)/(MSP/1000))

**Drivers of population differentiation in phenotypic plasticity in a temperate conifer: a 27-year study**

Raúl de la Mata, Rafael Zas, Gloria Bustingorri, Luis Sampedro, Marc Rust, Ana Hernández-Serrano, Anna Sala

**Table SI.2 Location and climate at the three test sites.**

Table SI.2 Location, main climatic features from planting to the assessment date (1974-2001 period) and overall means for diameter at age 27 of the *Pinus ponderosa* genetic trials.

|  | Condon |  | Little Wolf |  | Lubrecht |
| --- | --- | --- | --- | --- | --- |
| Longitude (W) | 113º 43’ 06” |  | 114º 57’ 09” |  | 113º 28’ 32” |
| Latitude (N) | 45º 32’ 38” |  | 48º 17’ 41” |  | 46º 53’ 15” |
| Elevation (a.s.l., m) | 1,130 |  | 997 |  | 1,260 |
| Diameter at breast height mean at age 27 (cm) | 18.7 |  | 17.8 |  | 15.5 |
| **Climate parameters (period 1974-2001)** |  |  |  |  |  |
| Mean annual temperature (MAT, ºC) | 6.2 |  | 5.8 |  | 6.2 |
| Mean temperature of the warmest month (MTWM, ºC) | 18.4 |  | 17.9 |  | 18.5 |
| Mean temperature of the coldest month (MTCM, ºC) | -5.4 |  | -6.0 |  | -5.5 |
| Mean annual precipitation (MAP, mm) | 561 |  | 533 |  | 451 |
| Mean summer precipitation (MSP, mm)^1^ | 120 |  | 110 |  | 115 |
| Summer Heat/Moisture Index (SHM)^2^ | 92.7 |  | 97.9 |  | 96.3 |

^1^ Rain fallen during the months of June, July and August

^2^ (Mean temperature of the warmest month) / (Mean summer precipitation/1000)

**Drivers of population differentiation in phenotypic plasticity in a temperate conifer: a 27-year study**

Raúl de la Mata, Rafael Zas, Gloria Bustingorri, Luis Sampedro, Marc Rust, Ana Hernández-Serrano, Anna Sala

**SI Methods 1. Causes of the population by site interaction.**

Spatially adjusted data were analyzed by fitting mixed models with site as a fixed factor, and population and population × site interaction as random factors (Crossa et al., 2004; Yang, 2002). The mixed models were fitted using the mixed procedure of SAS (Littell et al., 2006), accommodating the SAS programs of Yang (2002) to our experimental design. Variance components were estimated using the reml method. The estimation of the population covariance structure (variances and covariance across sites) was achieved by including the subject and type option in the random statement. Heterogeneity of residual variances across sites was implemented with the group option of the repeated statement.

In order to explore and interpret the Population × Environment interaction, different reduced models constraining different elements of the population covariance structure were fitted. Constraints to the population covariance structures were specified by choosing appropriate predefined covariance models for the type option in the random statement, and/or by fixing specific covariance parameters to certain values using the hold option of the parms statement (Crossa et al., 2004; Fry, 2004; Yang, 2002). Out-of-bond population correlations (> 1, or < -1) were avoided by including the upperb and lowerb option in the parms statement. A detailed list of the different models and the corresponding hypothesis analyzed is listed in Table SI.3. Hypothesis testing regarding the constraints imposed on the population-covariance structure was done by comparing the restricted log-likelihoods (RLL) of the constrained model and the unconstrained model (usually the full model with an unstructured population covariance structure, see later). Under the null hypothesis that the full covariance model is not different from the reduced covariance model, the log-likelihood ratio LLR = -2(RLLreduced model – RLLfull model) is distributed approximately as χ^2^ with degrees of freedom given by the difference between the number of covariance parameter specifying the full model and the reduced model (Fry, 2004).

**Table SI.3** Description of the full model and reduced models for testing different hypotheses on the interpretation of the population by environment interaction. The reduced models constrain different elements of the population variance-covariance structure^(1)^ by specifying in the random statement of proc mixed different types of covariance structures (CovStruc), and/or constraining different covariance parameters with the hold option of the parms statement^(2)^. The total number of parameters to be estimated (# parms) for analyses is also given. All models assume heterogeneity of residual variances across sites. All hypotheses are tested by comparing the reduced models with the full model, except the H1 hypothesis which is tested by comparing the H1 model *versus* the H3 model.

|  | |  |  |  |  |
| --- | --- | --- | --- | --- | --- |
| Model and hypothesis tested | | Constraints | CovStruc^(2)^ | Parameters to be estimated | # parms |
|  |  |  |  |  |  |
| H0 | Full model. All causes of population × environment interaction are allowed | None | UNR | $\sigma_{G1}^{2},\sigma_{G2}^{2},\sigma_{G3}^{2}$ $\rho_{12},\rho_{13},\rho_{23}$ $\sigma_{e1}^{2},\sigma_{e2}^{2},\sigma_{e3}^{2}$ | 9 |
|  |  |  |  |  |  |
| H1 | Homogeneity of population variance across sites |  | CS | $\sigma_{G}^{2},\sigma_{G\times E}^{2}$ $\sigma_{e1}^{2},\sigma_{e2}^{2},\sigma_{e3}^{2}$ | 5 |
|  |  |  |  |  |  |
| H2 | Perfect population correlations between all site pairs |  | CSH* | $\sigma_{G1}^{2},\sigma_{G2}^{2},\sigma_{G3}^{2}$ $\sigma_{e1}^{2},\sigma_{e2}^{2},\sigma_{e3}^{2}$ | 6 |
|  |  |  |  |  |  |
| H3 | Homogeneity of population covariance across all site pairs |  | CSH | $\sigma_{G1}^{2},\sigma_{G2}^{2},\sigma_{G3}^{2}$ $\rho$ $\sigma_{e1}^{2},\sigma_{e2}^{2},\sigma_{e3}^{2}$ | 7 |
|  |  |  |  |  |  |

^(1)^ Under the full model, the matrices for the family (Σ_G_) and error (Σ_e_) covariance structures are as follow:

$$\sum_{G} =\left[ \begin{matrix} \sigma_{G1}^{2} & \rho_{12} & \rho_{13} \\ \rho_{12} & \sigma_{G2}^{2} & \rho_{23} \\ \rho_{13} & \rho_{23} & \sigma_{G3}^{2} \end{matrix} \right]\quad\quad\quad\quad\sum_{e} =\left[ \begin{matrix} \sigma_{e1}^{2} & 0 & 0 \\ 0 & \sigma_{e2}^{2} & 0 \\ 0 & 0 & \sigma_{e3}^{2} \end{matrix} \right]$$

where σ^2^_Gi_ and σ^2^_ei_ are the population and residual variances in site *i*, and ρ_ij_ is the population correlation between site *i* and *j*.

^(2)^ Further constrains in specific elements of the variance-covariance matrix are denoted by an * in the CovStruc type.

We specifically tested for the contribution of each of the different possible causes of Population × Environment interaction. Homogeneity of population variances was analyzed by comparing the CS population covariance structure model (model H1 in Table SI.3) with the heterogeneous compound symmetry covariance structure model (CSH, model H3). Deviations from perfect correlations, which can be interpreted as a test of cross-over interactions (Yang, 2007), were analyzed by comparing a model in which all family correlations are fixed to 1 (model H2 in Table SI.3) with the full model H0. Finally, if significant deviations from perfect correlations were detected, we examined whether family correlations between sites were constant across all pairs of environments (model H3 in Table SI.3) or whether they differed depending on the site pairs (full model H0).

**Cited references**

Crossa, J., Yang, R.-C., & Cornelius, P. L. (2004). Studying crossover genotype × environment interaction using linear-bilinear models and mixed models. *J. Agric. Biol. Environ. Stat., 9*, 362-380.

Fry, J. D. (2004). *Estimation of genetic variances and covariances by restricted maximum likelihood using PROC MIXED*: A.M. Saxton (ed) Genetic analysis of complex traits using SAS. SAS Institute, Cary, NC, USA.

Littell, R. C., Milliken, G. A., Stroup, W. W., Wolfinger, R. D., & Schabenberger, O. (2006). *SAS System for mixed models, second edition*. Cary, NC, USA: SAS Institute.

Yang, R. C. (2002). Likelihood-based analysis of Genotype-Environment interactions. *Crop Science, 42*, 1434-1440. doi:https://doi.org/10.2135/cropsci2002.1434

Yang, R. C. (2007). Mixed-model analysis of crossover genotype-environment interactions. *Crop Science, 47*(3), 1051-1062. doi:https://doi.org/10.2135/cropsci2006.09.0611

**Drivers of population differentiation in phenotypic plasticity in a temperate conifer: a 27-year study**

Raúl de la Mata, Rafael Zas, Gloria Bustingorri, Luis Sampedro, Marc Rust, Ana Hernández-Serrano, Anna Sala

**SI Methods 2. Estimation of population plasticity.**

Plasticity estimates for every single population were obtained following the framework proposed in (Denis et al., 1997). Plasticity classical models can be readily embedded in a mixed model framework (Denis et al., 1997; Piepho, 1999) in which populations act as a fixed effect, and site and population by site interaction act as random effects. This framework represents a unified approach by which mixed models for multi-environment trial datasets can be expressed and compared. Each model is outlined as the sum of three components: the fixed terms, the random terms, and the residual term.

Mixed modelling allow to model the (S×P)*_ij_* term in a pretty flexible way (Denis et al., 1997), and classical stability approaches for describing such effect were handled using appropriate variance-covariance (vcov) structures as follows. The general forms of expectation (ε) and variance in our mixed model variants of Eq. (1) in main text are:

$$\varepsilon\left( Y_{j} \right)=\alpha_{j};var\left( Y \right)=\sigma_{S}^{2}+\tau+\sigma_{e}^{2}$$

where *α_j_* refers to the *j^th^* population main effect and *σ^2^_S_* and *σ^2^_e_* are the site variance and the error variance respectively. The term *τ* defines a particular vcov structure used to model the random term of interest *(S×P)_ij_* where *i* identifies the *i^th^* Site*,* and *j* the *j^th^* population . Four different vcov structures which represent five stability measures were fitted to population by site effects:

(1) Simple **(τ_1_)**: cov(*S×P_ij_*; *S×P_ij*_*) = *σ^2^_(S×P)_* when *j* = *j**, otherwise cov(*S×P_ij_*; *S×P_ij*_*) = 0.

(2) Diagonal **(τ_2_)**: cov(*S×P_ij_*; *S×P_ij*_*) = *σ^2^ _(S×P)i_* when *j* = *j**, otherwise cov(*S×P_ij_*; *S×P_ij*_*) = 0.

(3) Factor analytic 1 **(τ_3_)**: cov(*S×P_ij_*; *S×P_ij*_*) = *λ*_1j_ *λ*_1j*_ + *σ^2^_d_* when *j* = *j**, otherwise cov(*S×P_ij_*; *S×P_ij*_*) = *λ*_1j_ *λ*_1j*_.

Where *λ*_1j_ and *λ*_1j*_ are site-specific multiplicative parameters and *σ^2^_d_* is a common residual variance (Piepho, 1997).

(4) Factor analytic 1 + heterogeneity **(τ_4_)**: cov(*S×P_ij_*; *S×P_ij*_*) = *λ*_1j_ *λ*_1j*_ + ${\sigma^{2}}_{d_{k}}$ when *j* = *j**, otherwise cov(*S×P_ij_*; *S×P_ij*_*) = *λ*_1j_ *λ*_1j*_.

Where ${\sigma^{2}}_{d_{k}}$ accounts for residual heterogeneity (i.e., site-specific deviation; Piepho, 1997).

Hence, the stability models were as follows:

*Model 1* (additive mixed-effects model): This is the simplest model in which the variance takes the form:

$$var\left( Y \right)=\sigma_{S}^{2}+\tau_{1}+\sigma_{e}^{2}$$

Where *σ^2^_S_*, *σ^2^_e_* and *τ*_1_ are defined as above. According to this model the populations do not differ in stability.

*Model 2* (general heteroscedastic model): This model extends the additive model by attributing a different variance component (or stability measure) to each population:

$$var\left( Y \right)=\sigma_{S}^{2}+\tau_{2}+\sigma_{e}^{2}$$

This model is also known as Shukla’s stability variance (Shukla, 1972).

*Model 3* (Finlay-Wilkinson regression): This is the mixed model version of the broadly used Finlay-Wilkinson regression on the environmental mean (Finlay & Wilkinson, 1963), which can be fitted implementing a simplified factor analytic vcov structure with one factor:

$$var\left( Y \right)=\tau_{3}+\sigma_{e}^{2}$$

In this model, the site variance component *σ^2^_S_* is dropped from the vcov structure underlying the response variable *Y*. Given that this vcov structure is over-parameterized, an identifiability constraint needs to be imposed. Here we used the constraint *σ^2^_w_* = 1, as population *λ_j_*’s in *τ*_3_ represent sensitivities to a hypothetical underlying variable *w_m_* (Piepho, 1997, 1999). Whatever the constraint used, the relative magnitude of the values of *λ_j_*’s points the sensitivity to unobservable environmental conditions as measured by the environmental means (Piepho, 1998), which can also be interpreted in terms of the reaction norm slope as applied in evolutionary biology (Chevin et al., 2013).

*Model 4* (Eberhart-Russell regression): This is the mixed model equivalent to the Eberhart-Russell stability model (Eberhart & Russell, 1966), which extends the Finlay–Wilkinson regression to allow for heterogeneity in genotype by environment interaction variances. It can be fitted using a complete factor analytic vcov structure with one factor:

$$var\left( Y \right)=\tau_{4}+\sigma_{e}^{2}$$

Here, as in *Model 3*, the environmental variance component *σ^2^_S_* is dropped from the modelled variance and an identifiability constraint needs to be imposed. The interpretation of *λ_j_*’s is identical to the Finlay–Wilkinson model.

*Model 5* (AMMI-1 model): This is the mixed model version of the additive main effects and multiplicative interaction model with one multiplicative component (Kempton, 1984). As for *Model 3*, it can also be fitted using a complete factor analytic vcov structure with one factor. In this case, however, environmental variance component *σ^2^_S_* is retained for modelling purposes as follows:

$$var\left( Y \right)=\sigma_{S}^{2}+\tau_{3}+\sigma_{e}^{2}$$

Here, *λ*_1j_, as defined in *τ*_3_, is the factor loading associated with a particular population, which can be interpreted as the sensitivity of this population to the value of a hypothetical environmental variable (or factor score) for site *i* (Piepho, 1997; Smith et al., 2001).

The superiority of different vcov structure-based models was compared by computing information criteria such as Akaike’s information criterion (AIC) and Bayesian information criterion (BIC). Both involve a penalty for the number of parameters in the vcov structure, which favors parsimonious models, but BIC penalizes a large number of parameters more strongly than does AIC. Both statistics are in the smaller-is-better form.

The analyses were performed using the mixed procedure of SAS (Littell et al., 2006).

For model convergence purposes, population-site means was used as the response variable, the family effect was dropped from the model and homogeneity of residual variances across sites was assumed.

**Cited references**

Chevin, L. M., Collins, S., & Lefevre, F. (2013). Phenotypic plasticity and evolutionary demographic responses to climate change: taking theory out to the field. *Functional Ecology, 27*(4), 966-979. doi:https://doi.org/10.1111/j.1365-2435.2012.02043.x

Denis, J. B., Piepho, H. P., & VanEeuwijk, F. A. (1997). Modelling expectation and variance for genotype by environment data. *Heredity, 79*, 162-171. doi:https://doi.org/10.1038/sj.hdy.6881990

Eberhart, S. A., & Russell, W. A. (1966). Stability parameters for comparing varieties. *Crop Science, 6*(1), 36-40. doi:https://doi.org/10.2135/cropsci1966.0011183X000600010011x

Finlay, K. W., & Wilkinson, G. N. (1963). The analysis of adaptation in a plant breeding programme. *Australian Journal of Agricultural Science, 14*, 742-745.

Kempton, R. A. (1984). The use of biplots in interpreting variety by environment interactions. *Journal of Agricultural Science, 103*, 123-135. doi:https://doi.org/10.1017/s0021859600043392

Littell, R. C., Milliken, G. A., Stroup, W. W., Wolfinger, R. D., & Schabenberger, O. (2006). *SAS System for mixed models, second edition*. Cary, NC, USA: SAS Institute.

Piepho, H. P. (1997). Analyzing genotype-environment data by mixed models with multiplicative terms. *Biometrics, 53*(2), 761-766. doi:https://doi.org/10.2307/2533976

Piepho, H. P. (1998). Methods for comparing the yield stability of cropping systems - A review. *Journal of Agronomy and Crop Science, 180*(4), 193-213. doi:https://doi.org/10.1111/j.1439-037X.1998.tb00526.x

Piepho, H. P. (1999). Stability analysis using the SAS system. *Agronomy Journal, 91*(1), 154-160. doi:https://doi.org/10.2134/agronj1999.00021962009100010024x

Shukla, G. K. (1972). Some statistical aspects of partitioning genotype environmental components of variability. *Heredity, 29*, 237-245. doi:https://doi.org/10.1038/hdy.1972.87

Smith, A. B., Cullis, B. R., & Thompson, R. (2001). Analyzing Variety by Environment Data Using Multiplicative Mixed Models and Adjustments for Spatial Field Trend. *Biometrics, 57*, 1138-1147.

**Drivers of population differentiation in phenotypic plasticity in a temperate conifer: a 27-year study**

Raúl de la Mata, Rafael Zas, Gloria Bustingorri, Luis Sampedro, Marc Rust, Ana Hernández-Serrano, Anna Sala

**SI Methods 3. Estimation of environmental heterogeneity at seed source locations**.

Estimating environmental heterogeneity in a statistically robust manner has proven to be a challenge (White & Hood, 2004). The mosaics of heterogeneity and environmental variability can be captured and extracted by using geostatiscal approaches (Garrigues et al., 2006). Semivariograms are reportedly an efficient method to characterize the structure of spatial continuity (Guedes et al., 2015), due to their potential to describe the spatial variability of data. The semivariogram is a graphical representation of the spatial variability in a given set of data (Cohen et al., 1990). The relationship between a pair of locations within the landscape can be calculated with the variogram function (Equation SI.1), namely *2γ(h)*, which corresponds to the mathematical expectation of the squared difference between pairs of points separated by a distance *h*, where *Z(x)* is the value of the regionalized variable at point *x*, *Z(x + h)* is the value at *x + h*. The semivariogram function depends on the location *x*, and the distance between locations *h*.

2$\gamma\left( h \right)=E\left\{ \left[ Z\left( x \right)-Z\left( x+h \right) \right]^{2} \right\}$ [**SI.1**]

The experimental semivariogram is defined as the half of the average squared difference between values separated by a given lag, where this lag is a vector in distance (Atkinson & Lewis, 2000). It was estimated using Equation SI.2, where *γ(h)* is the estimator of the semivariance for each distance *h*, *N(h)* is the number of pairs of points separated by the distance *h*, *Z(x)* is the value of the measured variable at point *x* and *Z(x+h)* is the value at point *x+h*.

$\gamma\left( h \right)=\frac{1}{2N\left( h \right)}\sum_{i=1}^{N\left( h \right)} \left[ Z\left( x \right)-Z\left( x+h \right) \right]^{2}$ [**SI.2**]

Spatial variance versus distance *h* is the graphical representation of the semivariogram, which allows obtaining an estimate of the variance value for different combinations of pairs of points. The semivariogram is characterized by three parameters: *sill* (σ²), *range* (φ) and *nugget effect* (τ²; see Fig. SI.1). The *sill* or *max variance* parameter is the plateau reached by the semivariance values and shows the total spatial variation of the data. The *range* or *patch size* parameter is the distance at which the semivariogram reaches the *sill*, showing the distance at which the data cease to be correlated. The *nugget effect* is the combination of sampling errors and variations that happen at scales smaller than the distance between the sampled points (Curran, 1988).

**Fig. SI.1** Hypothetical standard semivariogram showing the observed semivariance for distance classes (dots) and the fitted model (solid line). Three parameters derived from the semivariogram characterize the spatial structure pattern: (a) the distance at which the asymptote begins (*φ*), which indicates the range or patch size of heterogeneity below which data are stochastically dependent, (b) the asymptote, called sill (*σ^2^*), which is an estimate of total variance, and (c) the nugget (*τ^2^*) or intercept at distance zero, which represents the variance due to sampling error and/or spatial dependence at scales not explicitly sampled. The larger the (*σ^2^*- *τ^2^*/ *σ^2^*) ratio, the greater the intensity of the spatial structure (*I*).

**
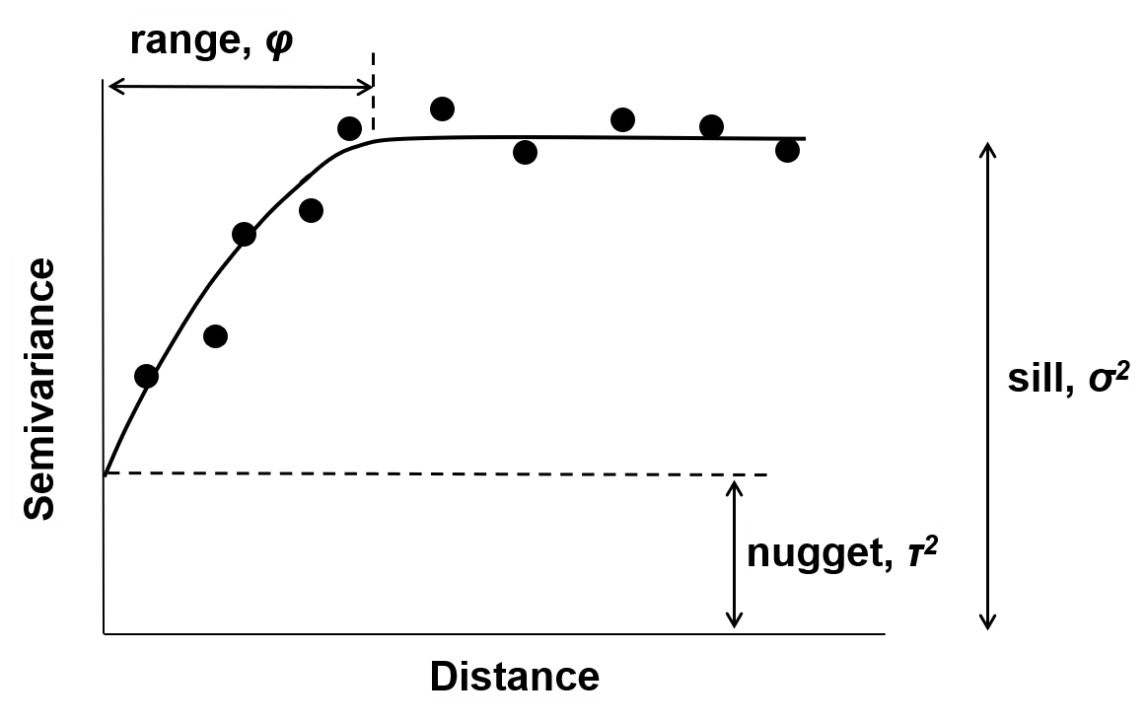
**

Here we took advantage of the *max variance* and *patch size* parameters as descriptors of the environmental heterogeneity at the seed source locations in terms of total variation and mosaic structure respectively (see Fig. SI.2). Hence, we constructed empirical semivariograms upon climate data around every seed source location using the variogram procedure in SAS (SAS-Institute, 2013). Thereafter, Spherical, Exponential or Gaussian models were fitted to the experimental semivariogram using the nlin procedure in SAS (see Table SI.4 for description of the models). Model selection was based on the goodness of fit (adjusted non-linear *R^2^*) between the empirical and theoretical semivariograms. *Max variance* (Table SI.5) and *patch size* (Table 4 in the main text) parameters for describing environmental heterogeneity were then extracted from the theoretical semivariogram models, taking into account that *patch size* for Spherical models is the *range* (φ), but φ×3 for Exponential models and φ×$\sqrt{3}$ for Gaussian models.

**Fig. SI.2** Contour plots for four types of spatial heterogeneity for a surface pattern and their respective hypothetical semivariograms: a) small patches of heterogeneity and large variation among patches is characterized by a short range and a large sill (*max variance*) in the semivariogram; b) small patches and reduced variation among patches is characterized by a short range and a small sill; c) large patches and large variation among patches is characterized by larger range and larger sill; and d) large patches and reduced variation among patches is characterized by a larger range and a smaller sill.

**
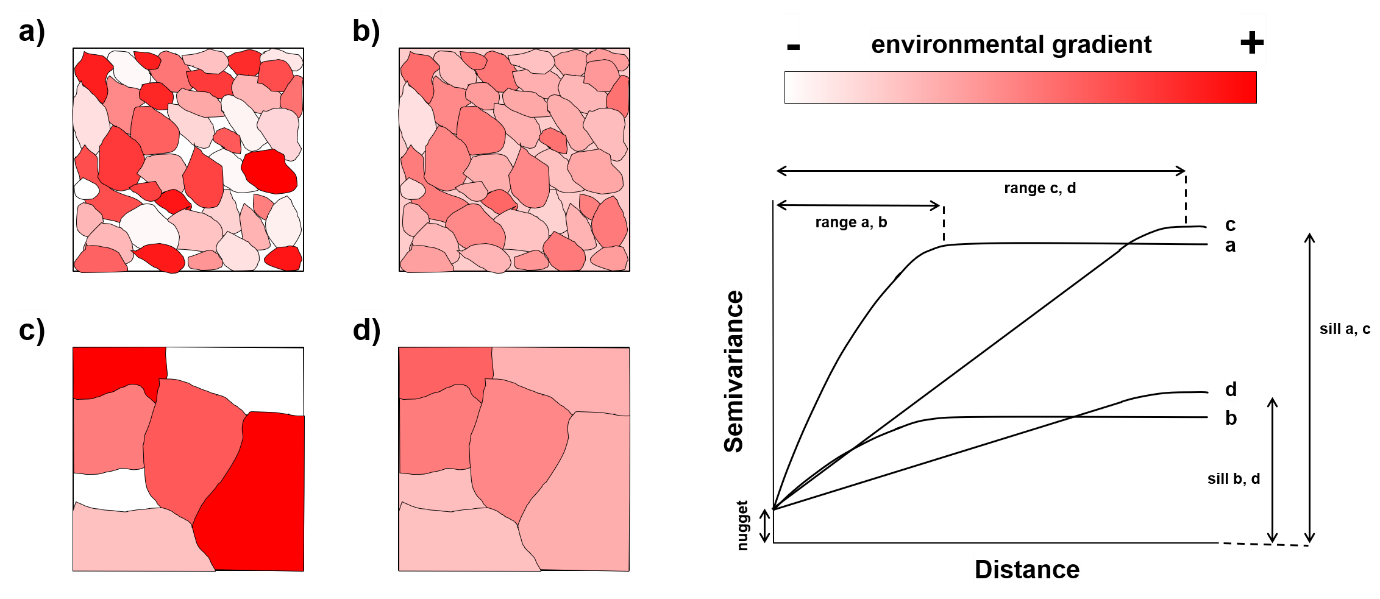
**

**Table SI.4** Models showing best goodness of fit statistics (adjusted non-linear *R^2^*) to the empirical semivariogram for every climate parameter in every population.

| **Population** | **MAT^1^** | **MTWM^2^** | **MTCM^3^** | **MAP^4^** | **MSP^5^** | **SHM^6^** |
| --- | --- | --- | --- | --- | --- | --- |
| **601** | Spherical**^7^** | Spherical | Gaussian**^8^** | Spherical | Exponential**^9^** | Spherical |
| **604** | Gaussian | Gaussian | Gaussian | Gaussian | Gaussian | Gaussian |
| **605** | Spherical | Spherical | Spherical | Spherical | Spherical | Spherical |
| **606** | Spherical | Spherical | Spherical | Exponential | Exponential | Gaussian |
| **612** | Spherical | Spherical | Spherical | Spherical | Spherical | Gaussian |
| **613** | Spherical | Spherical | Spherical | Gaussian | Spherical | Spherical |
| **615** | Gaussian | Gaussian | Gaussian | Gaussian | Gaussian | Gaussian |
| **626** | Spherical | Spherical | Gaussian | Gaussian | Gaussian | Gaussian |
| **629** | Spherical | Spherical | Spherical | Spherical | Spherical | Spherical |
| **630** | Spherical | Spherical | Spherical | Exponential | Spherical | Spherical |
| **631** | Spherical | Spherical | Gaussian | Gaussian | Spherical | Gaussian |
| **641** | Spherical | Spherical | Gaussian | Spherical | Spherical | Gaussian |
| **642** | Spherical | Spherical | Spherical | Spherical | Spherical | Gaussian |
| **665** | Spherical | Spherical | Exponential | Exponential | Spherical | Spherical |
| **666** | Spherical | Spherical | Gaussian | Spherical | Spherical | Spherical |
| **676** | Spherical | Spherical | Gaussian | Spherical | Spherical | Gaussian |
| **677** | Gaussian | Gaussian | Gaussian | Gaussian | Gaussian | Gaussian |
| **678** | Spherical | Spherical | Exponential | Gaussian | Gaussian | Spherical |
| **683** | Spherical | Spherical | Gaussian | Gaussian | Gaussian | Gaussian |
| **684** | Gaussian | Gaussian | Spherical | Gaussian | Gaussian | Gaussian |
| **685** | Spherical | Spherical | Gaussian | Spherical | Spherical | Spherical |
| **688** | Spherical | Spherical | Gaussian | Gaussian | Gaussian | Gaussian |
| **689** | Spherical | Spherical | Spherical | Spherical | Spherical | Spherical |

^1^ MAT: mean annual temperature

^2^ MTWM: mean temperature of the warmest month

^3^ MTCM: mean temperature of the coldest month

^4^ MAP: mean annual precipitation

^5^ MSP: mean annual summer (May to Sept.) precipitation

^6^ SHM: summer heat-moisture index

^7^ Spherical model: $\gamma\left( h \right)=\left\{ \sigma^{2}\left[ \frac{3}{2}\left( \frac{h}{\varphi} \right)-\frac{1}{2}\left( \frac{h}{\varphi} \right)^{3} \right] \right\}$, where *γ(h)*=semivariance; *h*=distance; *σ^2^*=sill and *φ*=range.

^8^ Gaussian model: $\gamma\left( h \right)=\sigma^{2}\left[ 1-e^{\left[ -3\left( \frac{h}{\varphi} \right)^{2} \right]} \right]$, where *γ(h)*=semivariance; *h*=distance; *σ^2^*=sill and *φ*=range.

^9^ Exponential model: $\gamma\left( h \right)=\sigma^{2}\left[ 1-e^{\left[ -3\left( \frac{h}{\varphi} \right) \right]} \right]$, where *γ(h)*=semivariance; *h*=distance; *σ^2^*=sill and *φ*=range.

**Table SI.5** Max variance (sill, *σ²*) of environmental heterogeneity derived from the theoretical fitted semivariograms for every climate parameter in every population.

| **Population** | **MAT^1^** | **MTWM^2^** | **MTCM^3^** | **MAP^4^** | **MSP^5^** | **SHM^6^** |
| --- | --- | --- | --- | --- | --- | --- |
| **601** | 1.761 | 2.290 | 0.901 | 9622.5 | 3644.8 | 343.42 |
| **604** | 7.689 | 11.904 | 1.360 | 335441.7 | 18778.7 | 1362.57 |
| **605** | 1.011 | 1.450 | 0.310 | 11340.2 | 856.1 | 252.63 |
| **606** | 1.042 | 1.547 | 0.322 | 9410.3 | 912.2 | 173.15 |
| **612** | 1.263 | 2.260 | 0.558 | 42362.9 | 1130.9 | 370.38 |
| **613** | 0.582 | 2.676 | 0.815 | 6086.4 | 741.2 | 94.29 |
| **615** | 13.494 | 15.786 | 9.801 | 483310.8 | 16212.3 | 1308.63 |
| **626** | 4.601 | 4.282 | 3.494 | 527256.7 | 36452.0 | 590.70 |
| **629** | 2.008 | 2.874 | 0.701 | 24109.0 | 962.9 | 404.12 |
| **630** | 0.774 | 1.177 | 0.268 | 12144.3 | 653.8 | 176.37 |
| **631** | 2.227 | 2.875 | 0.698 | 52035.9 | 4549.8 | 473.98 |
| **641** | 2.933 | 3.510 | 1.173 | 20373.8 | 1641.5 | 424.77 |
| **642** | 0.599 | 0.658 | 0.399 | 27802.0 | 1742.5 | 171.22 |
| **665** | 0.562 | 0.964 | 0.151 | 48475.6 | 2764.6 | 373.23 |
| **666** | 0.183 | 0.225 | 0.362 | 13005.0 | 1033.7 | 220.61 |
| **676** | 3.741 | 4.667 | 2.065 | 186205.3 | 15366.2 | 419.11 |
| **677** | 3.712 | 4.865 | 1.851 | 152888.2 | 5113.8 | 489.42 |
| **678** | 0.228 | 0.282 | 0.151 | 2559.4 | 267.8 | 72.96 |
| **683** | 2.058 | 2.548 | 0.811 | 43389.1 | 3139.1 | 423.48 |
| **684** | 2.698 | 4.521 | 0.215 | 347981.9 | 26122.9 | 834.02 |
| **685** | 1.662 | 2.215 | 0.585 | 16133.1 | 480.4 | 255.12 |
| **688** | 3.405 | 4.572 | 1.256 | 60113.1 | 3581.8 | 503.45 |
| **689** | 2.293 | 3.352 | 0.867 | 50345.6 | 2057.2 | 359.76 |
| **Coefficient of variation across populations** | 1.11 | 1.01 | 1.59 | 1.47 | 1.48 | 0.75 |

^1^ MAT: mean annual temperature (ºC^2^)

^2^ MTWM: mean temperature of the warmest month (ºC^2^)

^3^ MTCM: mean temperature of the coldest month (ºC^2^)

^4^ MAP: mean annual precipitation (mm^2^)

^5^ MSP: mean annual summer (May to Sept.) precipitation (mm^2^)

^6^ SHM: summer heat-moisture index

**Cited references**

Atkinson, P. M., & Lewis, P. (2000). Geostatistical classification for remote sensing: an introduction. *Computers & Geosciences, 26*(4), 361-371. doi:https://doi.org/10.1016/s0098-3004(99)00117-x

Cohen, W. B., Spies, T. A., & Bradshaw, G. A. (1990). Semivariograms of digital imagery for analysis of conifer canopy structure. *Remote Sensing of Environment, 34*(3), 167-178. doi:https://doi.org/10.1016/0034-4257(90)90066-u

Curran, P. J. (1988). The semivariogram in remote sensing: an introduction. *Remote Sensing of Environment, 24*(3), 493-507. doi:https://doi.org/10.1016/0034-4257(88)90021-1

Garrigues, S., Allard, D., Baret, F. E., & Weiss, M. (2006). Quantifying spatial heterogeneity at the landscape scale using variogram models. *Remote Sensing of Environment, 103*(1), 81-96.

Guedes, I. C. D. L., Mello, J. M. D., Silveira, E. M. D. O., Mello, C. R. D., Reis, A. A. D., & Gomide, L. R. (2015). Continuidade espacial de características dendrométricas em povoamentos clonais de *Eucalyptus* sp. avaliada ao longo do tempo. *Cerne, 21*(4), 527-534.

SAS-Institute. (2013). *SAS® 9.4 Statements: Reference*. Cary, NC: SAS Institute Inc.

White, D. A., & Hood, C. S. (2004). Vegetation patterns and environmental gradients in tropical dry forests of the northern Yucatan Peninsula. *Journal of Vegetation Science, 15*(2), 151-160. doi: <https://doi.org/10.1111/j.1654-1103.2004.tb02250.x>

**Drivers of population differentiation in phenotypic plasticity in a temperate conifer: a 27-year study**

Raúl de la Mata, Rafael Zas, Gloria Bustingorri, Luis Sampedro, Marc Rust, Ana Hernández-Serrano, Anna Sala

**Fig. SI.3 Overall mean of height and diameter at breast height growth over time in each of the test sites.**


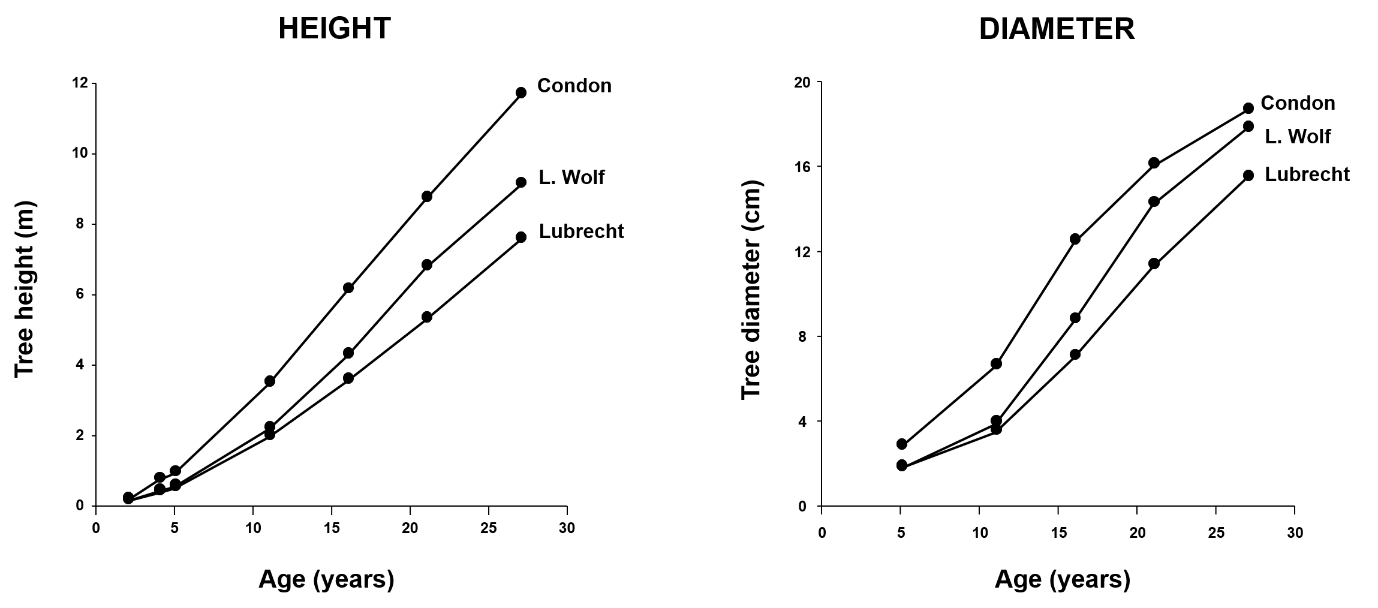

Supplement: Supplementary file 1 — Appendix S1 [file EVA-15-1945-s001.docx]
